# Genome analysis and genome-wide proteomics of *Thermococcus gammatolerans*, the most radioresistant organism known amongst the Archaea

**DOI:** 10.1186/gb-2009-10-6-r70

**Published:** 2009-06-26

**Authors:** Yvan Zivanovic, Jean Armengaud, Arnaud Lagorce, Christophe Leplat, Philippe Guérin, Murielle Dutertre, Véronique Anthouard, Patrick Forterre, Patrick Wincker, Fabrice Confalonieri

**Affiliations:** 1 Laboratoire de Génomique des Archae, Université Paris-Sud 11, CNRS, UMR8621, Bât400 F-91405 Orsay, France; 2 CEA, DSV, IBEB Laboratoire de Biochimie des Systèmes Perturbés, Bagnols-sur-Cèze, F-30207, France; 3 CEA, DSV, Institut de Génomique, Genoscope, rue Gaston Crémieux CP5706, F-91057 Evry Cedex, France; 4 Laboratoire de Biologie moléculaire du gène chez les extrêmophiles, Université Paris-Sud 11, CNRS, UMR8621, Bât 409, F-91405 Orsay, France

## Abstract

**Background:**

*Thermococcus gammatolerans *was isolated from samples collected from hydrothermal chimneys. It is one of the most radioresistant organisms known amongst the Archaea. We report the determination and annotation of its complete genome sequence, its comparison with other Thermococcales genomes, and a proteomic analysis.

**Results:**

*T. gammatolerans *has a circular chromosome of 2.045 Mbp without any extra-chromosomal elements, coding for 2,157 proteins. A thorough comparative genomics analysis revealed important but unsuspected genome plasticity differences between sequenced *Thermococcus *and *Pyrococcus *species that could not be attributed to the presence of specific mobile elements. Two virus-related regions, tgv1 and tgv2, are the only mobile elements identified in this genome. A proteogenome analysis was performed by a shotgun liquid chromatography-tandem mass spectrometry approach, allowing the identification of 10,931 unique peptides corresponding to 951 proteins. This information concurrently validates the accuracy of the genome annotation. Semi-quantification of proteins by spectral count was done on exponential- and stationary-phase cells. Insights into general catabolism, hydrogenase complexes, detoxification systems, and the DNA repair toolbox of this archaeon are revealed through this genome and proteome analysis.

**Conclusions:**

This work is the first archaeal proteome investigation done at the stage of primary genome annotation. This archaeon is shown to use a large variety of metabolic pathways even under a rich medium growth condition. This proteogenomic study also indicates that the high radiotolerance of *T. gammatolerans *is probably due to proteins that remain to be characterized rather than a larger arsenal of known DNA repair enzymes.

## Abstract 2

The genome sequence of Thermococcus gammatolerans, a radioresistant archaeon, is described; a proteomic analysis reveals that radioresistance may be due to unknown DNA repair enzymes.

## Background

Thermococcales are strictly anaerobic and hyperthermophilic archaea belonging to the Euryarchaeota phylum. In this order, three genera are distinguished: *Pyrococcus *[1], *Thermococcus *[2] and *Palaeococcus *[3]. With about 180 different species listed to date, the *Thermococcus *genus is the largest archaeal group characterized so far. They have been isolated from terrestrial hot springs, deep oil reservoirs, and are widely distributed in deep-sea environments [4,5]; they are considered as key players in marine hot-water ecosystems. *Thermococcus *species are able to grow anaerobically on various complex substrates, such as yeast extract, peptone, and amino acids in the presence of elemental sulfur (S°), and yield hydrogen sulfide. Several species are also capable of fermenting peptides, amino acids or carbohydrates without sulfur producing acids, CO_2 _and H_2 _as end products [6,7]. Recently, some species such as *Thermococcus *strain AM4 and *Thermococcus onnurineus *NA1 were shown to be capable of lithotrophic growth on carbon monoxide [8,9]. In this case, the CO molecule, probably oxidized into CO_2_, is used as energy and/or carbon source.

Five Thermococcales genomes have been sequenced and annotated so far: *Pyrococcus horikoshii *[10], *Pyrococcus furiosus *[11], *Pyrococcus abyssi *[12], *Thermococcus kodakaraensis *KOD1 [13] and *T. onnurineus *NA1 [8]. Although their respective gene contents are highly conserved, synteny analyses have shown an extensive frequency of genomic DNA rearrangements in Thermococcales [14,15]. The relatively low fraction of insertion sequence elements or repeats in *Thermococcus *genomes contrasts with the fact that genome rearrangements are faster than normal protein sequence evolution [13].

Some hydrothermal chimneys in which many thermophilic prokaryotes were isolated were shown to be especially rich in heavy metals [16,17] and exposed to natural radioactivity doses hundreds of times higher than those found on the Earth's surface [18]. Although such extreme conditions were likely to have been much more common during the first stages of life on Earth, they are deleterious and few data are currently available regarding the strategies that thermophiles use to live in such environments. The hyperthermophilic archaeon *Thermococcus gammatolerans *was recently isolated from samples collected from hydrothermal chimneys located in the mid-Atlantic Ridge and at the Guyamas basin [19,20]. *T. gammatolerans *EJ3 was obtained by culture enrichment after irradiation with gamma rays at massive doses (30 kGy). It was described as an obligatory anaerobic heterotroph organism that grows optimally at 88°C in the presence of sulfur or cystine on yeast extract, tryptone and peptone, producing H_2_S. This organism withstands 5 kGy of radiation without any detectable lethality [21]. Exposure to higher doses slightly reduces its viability whereas cell survival of other thermophilic radioresistant archaea drastically decreases when cells are exposed to such radiation doses [20]. Based on these data, *T. gammatolerans *is one of the most radioresistant archaeon isolated and characterized thus far. As Archaea and Eukarya share many proteins whose functions are related to DNA processing [22], the radioresistant *T. gammatolerans *EJ3 species is a unique model organism along the Archaea/Eukarya branch of the phylogenetic tree of life. In contrast to the well-characterized *Deinococcus radiodurans*, the radioresistant model amongst Bacteria [23,24], the lack of knowledge on *T. gammatolerans *EJ3 urges us to further characterize this archaeon using the most recent OMICs-based methodologies.

Although more than 50 archaeal genomes have been sequenced so far, only a few archaea have been analyzed in depth at both the genome and proteome levels. *Halobacterium *sp. NRC-1 was the first archaeon to be analyzed for its proteome on a genome-wide scale. A partial proteome shotgun revealed 57 previously unannotated proteins [25]. A set of 412 soluble proteins from *Methanosarcina acetivorans *was identified with a two-dimensional gel approach [26]. In *Aeropyrum pernix *K1, 19 proteins that were not previously described in the genomic annotation were discovered [27]. *Halobacterium salinarum *and *Natronomonas pharaonis *proteomes were scrutinized with a special focus on amino-terminal peptides or low molecular weight proteins [28-30]. Although labor-intensive, proteogenomic re-annotation of sequenced genomes is currently proving to be very useful [31]. Moreover, genome-scale proteomics reveals whole proteome dynamics upon changes in physiological conditions.

Here we present a genome analysis of *T. gammatolerans *EJ3 and a detailed comparison with other Thermococcales genomes. To gain real insights into the physiology of *T. gammatolerans*, we analyzed the proteome content of exponential- and stationary-phase cells by a liquid chromatography (LC)-tandem mass spectrometry (MS/MS) shotgun approach and semi-quantification by spectral counting. *T. gammatolerans *is the first archaeon whose genome and proteome have been analyzed jointly at the stage of primary annotation. With these results in hand and its remarkable radiotolerance, *T. gammatolerans *is now a model of choice amongst the Archaea/Eukarya lineage.

## Results and discussion

### Genome sequence

The complete genome sequence of *T. gammatolerans *has been determined with good accuracy, with final error rate levels of less than 2.4 × 10^-05 ^before manual editing of 48 remaining errors. It is composed of a circular chromosome of 2,045,438 bp without extra-chromosomal elements, and a total of 2,157 coding sequences (CDSs) were identified (Table S1 in Additional data file 1). Their average size is 891 nucleotides, comprising CDSs ranging from 32 (tg2073, encoding a conserved hypothetical protein) to 4,620 amino acids (tg1747, encoding an orphan protein).

### Genome annotation accuracy as evaluated by proteomics

We analyzed the proteome content of *T. gammatolerans *grown in optimal conditions (rich medium supplemented with S°) at two stages, exponential and stationary. Total proteins were resolved by one-dimensional SDS-PAGE and identified by nanoLC-MS/MS shotgun analysis. From the large corpus of MS/MS spectra (463,840) that were acquired, 170,790 spectra could be assigned to 11,056 unique peptides (Table S6 in Additional data file 2). A total of 951 proteins were identified with very stringent search parameters (at least two peptides with *P *< 0.001; Table S7 in Additional data file 2). Our experimental results clearly show that all MS/MS identified peptides map to an entry in both the TGAM_ORF0 and TGAM_CDS1 databases (see Materials and methods), corresponding to 44% of the theoretical proteome and to a polypeptide coverage of 33% on average. Accordingly, all confident MS/MS spectra protein assignments confirmed the predicted genes, but we cannot exclude that a few new genes encoding small and non-abundant proteins may be present as such polypeptides typically resulted in a limited number of trypsic peptides that can be difficult to detect. While 45% of the theoretical proteome, composed of proteins ranging between 10 and 40 kDa, is detected by mass spectrometry, only 23% of proteins below 10 kDa are detected. This strong bias indicates that there may be some doubt regarding the real existence of some short annotated genes. Alternatively, most of them may correspond to non-abundant proteins.

### Translation start codon verification by mass spectrometry and amino-terminal modifications

After checking for trypsin and semi-trypsin specificities, we found 290 different amino-terminal peptidic signatures (Table S9 in Additional data file 3). They correspond to 173 different proteins. The start codon of 20 genes was incorrectly predicted and was corrected. Out of the 173 proteins, 70 exhibit a methionine at their amino terminus, 98 start with another amino acid, and 5 are found in both forms (Table S10 in Additional data file 3). The pattern for initial methionine cleavage is standard and depends on the steric hindrance of the second amino acid residue. As a result, polypeptides start with Ala (29 cases), Gly (18 cases), Pro (14 cases), Ser (12 cases), Thr (12 cases) and Val (18 cases).

A restricted set (13%) of these proteins (23 of 173) were found acetylated at their amino-terminal residue (Table S10 in Additional data file 3). This post-translational modification occurs for both cytosolic and membrane proteins. In contrast to halophilic organisms [32], we found in *T. gammatolerans *that the presence of an acidic amino acid (mainly Glu) in the second (when Met is not removed) or the third position of the polypeptide (when Met is removed) enhances the acetylation process (8 cases out of 11, and 10 cases out of 12, respectively). However, such a pattern does not imply acetylation as 25 proteins were found exclusively unacetylated. Remarkably, both acetylated and unacetylated amino termini were detected in 11 cases. In eukaryotes, three amino-terminal acetyltransferases, NatA, NatB, and NatC, have been described with preferential substrates [33]. We did not find any homologues of these acetyltransferase complexes in the *T. gammatolerans *genome but did find three putative N-acetyltransferases encoded by tg0455, tg1315, and tg1588. From the amino-terminal peptidic signatures that were recorded in our shotgun analysis, we deduced that *T. gammatolerans *encodes at least a functional analogue of NatA, because acetylation occurs on Ala, Gly, and Ser residues when the amino-terminal Met is removed (12 cases out of 12 different acetylated proteins), and a functional analogue of NatB that acetylates the Met residue when a Met-Glu, Met-Asp, or Met-Met dipeptide is located at the amino terminus of the protein. Such dipeptides are found for 9 out of 11 acetylated proteins; the remaining 2 acetylated proteins start with Met-Gln.

### Genome features

Table 1 summarizes the general features of *T. gammatolerans *compared with those of other sequenced Thermococcales. No significant differences in gene composition statistics were seen for these genomes. Amongst Thermococcales, a specific trait of *Thermococcus *genomes was noted when comparing the GC percentages of coding and inter-gene regions: this difference rises to 10% for *Thermococcus *compared to about 5% for *Pyrococcus*. As expected, average CDS identity values reflect the phylogenetic distance relationships within Thermococcales.

**Table 1:** General features of the six sequenced Thermococcales species*

	*T. gammatolerans*	*T. onnurineus*	*T. kodakaraensis*	*P. abyssi*	*P. horikoshii*	*P. furiosus*
Genome size (nt)	2,045,438	1,847,607	2,088,737	1,765,118	1,738,505	1,908,256
Percentage coding regions	94.0%	91.7%	93.2%	93.1%	95.0%	93.8%
GC%	53.6%	51.2%	52.0%	44.7%	41.9%	40,80%
Intergene GC%	43.3%	42.4%	42.0%	39.6%	39.8%	35.8%
Number of CDSs	2,157	1,976	2,306	1,896	1,955	2,125
Gene overlaps^†^	237 (11%)	402 (20%)	557 (24%)	317 (17%)	712 (36%)	657 (31%)
Mean CDS length (nt)	891	857	844	918	854	842
Average CDS identity with *T. gammatolerans*%^‡^	100%	76.7%	77.2%	72.8%	71.2%	71.5%
tRNAs	46	46	46	46	46	46
rRNAs	2× 5S, 7S, 16S, 23S	2× 5S, 7S, 16S, 23S	2× 5S, 7S, 16S, 23S	2× 5S, 7S, 16S, 23S	2× 5S, 7S, 16S, 23S	2× 5S, 7S, 16S, 23S

*Data for the five Thermococcales strains were from GenBank. ^†^Total number of overlaping genes and fraction of genes with overlaps (percentage in parentheses). ^‡^This refers to average identity percent values obtained by similarity matches with BLASTP. Nt, nucleotides.

*T. gammatolerans *shares 1,660 genes with *T. kodakaraensis *KOD1 whereas only 1,489 genes were found to be common with *T. onnurineus *NA1, a number similar to that obtained when *T. gammatolerans *is compared to *Pyrococcus *species. This result is due to the lower size of the *T. onnurineus *NA1 genome, which is about 200 kb shorter than the other sequenced *Thermococcus *genomes. Consequently, the three *Thermococcus *genomes share only 1,416 common genes (Table S2 in Additional data file 1). Remarkably, two-thirds of the 74 genes conserved in *T. gammatolerans *and *T. onnurineus *NA1 but missing in *T. kodakaraensis *KOD1 encode putative hydrogenase complexes that are present in several copies in *T. gammatolerans *and *T. onnurineus *NA1 genomes, or encode conserved proteins of unknown function. Among the six Thermococcales genomes, 1,156 genes are conserved (Table S3 in Additional data file 1). They were obviously present in the common ancestor before the divergence of *Thermococcus *and *Pyrococcus*. After searching for sequence similarities and specific motifs and domains in public databases, as defined in the Materials and methods, we are able to propose a function for 1,435 *T. gammatolerans *CDSs. Among the 722 remaining genes encoding hypothetical proteins, 214 are conserved in all the six sequenced Thermococcales. The products of one-sixth (120) of these genes were experimentally detected by our proteomic detection approach. *T. gammatolerans *possess a set of 326 genes absent in other sequenced Thermococcales (Table S4 in Additional data file 1). Among them, 98 are distributed in diverse functional categories as predicted by sequence similarity, the most important features being discussed below.

### Paradoxical genome plasticity in Thermococcales

**Figure 1:**
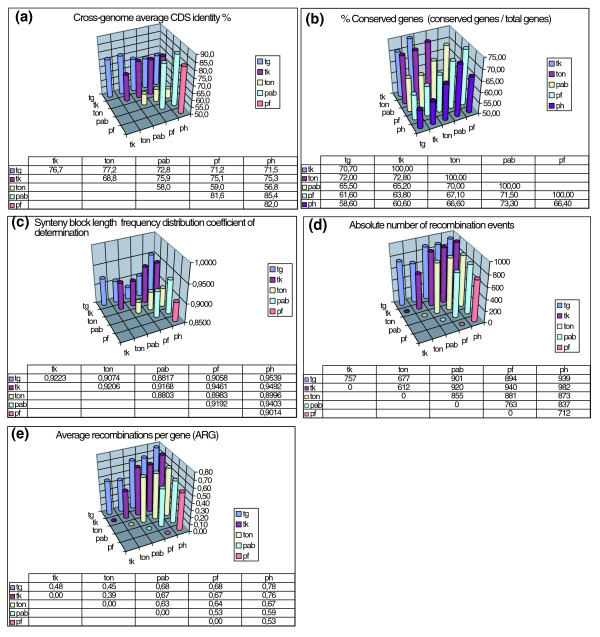
Thermococcales genome parameters defined in this study. For each parameter, a chart for genome pairs (tg, *T. gammatolerans*; tk, *T. kodakaraensis*; ton, *T. onnurineus*; pab, *P. abyssi*; pf, *P. furiosus*; ph, *P. horikoshii*) is shown in the upper part of the panel, and a table of data used to build the chart is shown in the lower part of the panel. **(a) **Cross-genome average CDS identity. Values were determined by compiling identity percentage of each gene first hit in a BLASTP full genome cross-match, using 80% alignment length and 0.3 of maximum bit score threshold values (see Materials and methods). Values were then averaged by the total number of similar genes in each pair. **(b) **Percentage of similar (conserved) genes for each genome pair. Numbers of similar genes were determined as in (a). The number of conserved genes in each genome pair was then averaged by half of the sum of the total number of genes from both genomes. **(c) **Genome pair values of least squares line of best fit determination coefficients (R^2^) for synteny block length distribution (Figure 2, left bottom). **(d) **Total number of recombination events for genome pairs. These numbers are actually the total number of synteny blocks + 1 within each genome pair. **(e) **Average recombinations per gene (ARG) for genome pairs. The total number of recombination values (from (d)) was normalized by the number of conserved genes in each pair.

The six closely related and fully sequenced Thermococcales species (three *Thermococcus*, *T. gammatolerans*, *T. kodakaraensis*, and *T. onnurineus*, and three *Pyrococcus*, *P. abyssi*, *P. horikoshii*, and *P. furiosus*) enable insights into ongoing genome evolution at a global scale since limited sequence divergence enables the fate of most genes in each considered lineage to be specifically tracked (Table 1 and Figure 1a). Most rearrangement mechanisms identified so far are non-random (for example, symmetry for replication-linked recombinations [34,35], site specificity for mobile elements [15,36,37], and recombination hotspots). For example, uneven fragmentation rates were described in archaea from pairwise comparisons at replication termini regions of *Pyrococcus *species [38], a situation already noted for bacterial genomes [39], although this does not preclude that random recombination prevails on a global genome scale. Determination of the chronology of genome recombination events among the three *Pyrococcus *species showed that, as a consequence, nucleotidic sequences can evolve at increased rates [15]. Here, we take advantage of the very high fraction of conserved genes between six Thermococcales (approximately 58 to 73%; Figure 1b) to deduce the global number of reciprocal recombination events and their distribution patterns.

**Figure 2:**
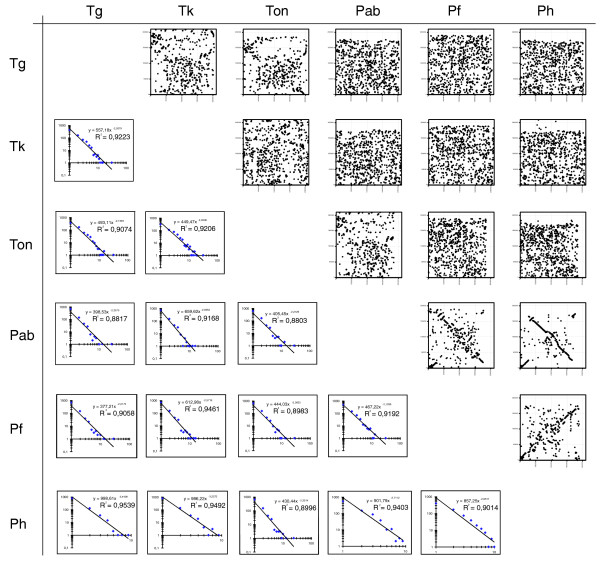
*Thermococcus *synteny analyses. Genome pair scatter plots are shown in the upper right. Similar genes (see Materials and methods) between all genome pairs (Tg, *T. gammatolerans*; Tk, *T. kodakaraensis*; Ton, *T. onnurineus*; Pab, *P. abyssi*; Pf, *P. furiosus*; Ph, *P. horikoshii*) were determined and their respective location on both genomes was plotted. Each dot represents a single gene. Coordinates are in nucleotides. Genome pair synteny block length frequency distributions are shown in the bottom left part. Synteny blocks within each genome pair were compiled, and their length frequency distributions were plotted on a log-log graph. In each plot, the equation of the least squares line of best fit is displayed, as well as the determination coefficient (R^2^) of the linear regression.

Pairwise genome scatter plots were determined to analyze recombination patterns between genomes. They show two different types of pattern (Figure 2, upper right), one in which chromosomes co-linearity is recognizable (see *Pyrococcus *pairs pab/ph/, pab/pf and ph/pf plots), and another where all genes seem randomly scattered, except for a few islands of syntenic blocks (see *Pyrococci*/*Thermococci *pairs plots: tg, tk, ton *versus *pab, ph, pf, and *Thermococcus *pairs: tg/tk, tg/ton and tk/ton). This is unexpected for *Thermococcus *pairs, since the overall number of similar genes is very close in *Thermococcus *and *Pyrococcus *species (approximately 71 to 73% and approximately 67 to 73%, respectively; Figure 1b), and their sequence similarity is very high (intra-*Thermoccocus *identity range 69 to 77%; intra-*Pyrococcus *identity range 81 to 85%; Figure 1a).

In order to determine the global recombination trends, we modeled a chromosome as a finite length segment disrupted by N hits (recombination events), each hit generating N + 1 intervals (fragments or synteny blocks) whose size frequency distribution obeys a power law when hits are at random (in which Frequency = a × Fragment_size^b^, a and b being constants). We determined synteny block length distribution for every genome pair (Figure 2, bottom left), and, in all cases, real distributions can be fitted to a power law model with good statistical support (R^2 ^range 0.88 to 0.95; coefficients of determination given by least square regression analysis; Figure 1c). We conclude that all six Thermococcales genomes exhibit random recombination distribution over the entire genome. Although unlikely, it could result from the summation of several local and mutually compensating recombination hot spots/regions, but there is no evidenced for this at this resolution. If we equate fragments of the model with real synteny blocks, the random hits hypothesis allows us to determine the number of recombination events yielding the observed distributions by summing the number of synteny blocks (minus 1). The absolute number of recombination events (Figure 1d) spans a rather narrow range (612 to 982 overall hits), which increases slightly when comparing intra- and inter-genus recombination frequencies (intra-*Thermococcus *hits 612 to 757; intra-*Pyrococcus *hits 712 to 837; *Pyrococcus*/*Thermococcus *855 to 982). We further normalized these values to cope with the number of conserved genes in each genome pair, and defined the average number of recombinations per gene (ARG) as ARG = Total number of recombination events/Number of conserved gene pairs in each genome pair. The overall ARG range is greater then before (0.39 to 0.78; Figure 1e) but, as expected, intra-genus ranges remained narrow (intra-*Thermococcus *ARG = 0.39 to 0.48; intra-*Pyrococcus *ARG = 0.53 to 0.59; *Pyrococcus*/*Thermococcus *ARG = 0.63 to 0.78). These results uncover a paradox, as smaller intra-*Thermococcus *ARG values correspond to more dispersed plots than higher intra-*Pyrococcus *ARG values. While an accurate measure of gene dispersion in pairwise genome comparisons is not yet at hand, it seems undeniable that high gene dispersion patterns are a consequence of the smaller ARG ratios among *Thermococci*. As a control, we determined the ARG ratios and scatter plots for three sequenced *Sulfolobus *species (*S. solfataricus P2*, *S. tokodai *and *S. acidocaldarius*; data not shown). In this case, very high ARG ratios ranging from 0.81 to 0.91 were obtained (R^2 ^range 0.95 to 0.96), although colinear regions on scatter plots could still be distinguished between genome pairs.

To help explain this paradox, the integrity of the *T. gammatolerans *chromosome can be questioned, since this strain has been isolated after gamma ray irradiation of 30 kGy. Several lines of evidence indicate that the chromosome did not undergo notable rearrangements: first, chromosome reconstitution kinetics from 2.5 kGy up to 7.5 kGy never show any alteration of the restriction patterns of repaired chromosomes (this work, [21] and not shown); second, its genome sequence does not exhibit any significant error rate in terms of number of frameshifts as well as pseudo-genes; third, nucleotidic cumulative compositional biases of AT nucleotides at the third codon position (AT3 skew as defined in [15]) display regular, nearly unperturbed patterns (data not shown); and fourth, scatter plot patterns of the two other *Thermococcus *species show that their recombination fate is identical to that of *T. gammatolerans*. Altogether, these data rule out the possibility that this behavior of *T. gammatolerans *is an artifact, and substantiate that chromosomal shuffling in *Thermococcus *species functions in a different mode than that in *Pyrococcus *and *Sulfolobus*, the last two presumably behaving in the expected way. As the decay of inter-species chromosome colinearity should be a progressive process under random conditions, long-range synteny should remain visible even for extended rates of divergence.

Whether the peculiar chromosome shuffling behavior of the Thermococci has any relation to the radiation-tolerance of *T. gammatolerans *is not known at present, but a group of 100 genes found in all *Thermococcus *species and absent from all *Pyrococcus *species (Table S5 in Additional data file 1) could be involved in this phenotype, as well as some specific genome nucleotidic compositional biases. We searched for ubiquitous oligonucleotide motifs that could act in the same way as Chi motifs, which influence double-stand break repair in the RecBCD pathway [40,41]. Such items are characterized by global over-representation and extended scattering across the chromosome because their function depends on a statistical significance. Although identification of new motifs remains challenging [42], if such motifs are present in *Thermococcus*, they must be absent in *Pyrococcus*, or *vice versa*. Indeed, we found two candidate octamers corresponding to these criteria: AGCTCCTC is the most overrepresented motif in 2 out of 3 *thermococci*, and the third most overrepresented in the other (third) one. TCCCAGGA is the third most overrepresented motif in one *pyroccoccus*, the fifth most overrepresented in another *pyrococcus *and the tenth most overrepresented motif in the third *pyrococcus*. Further characterization of these genes and sequences should now be undertaken to elucidate their roles and the molecular mechanisms associated with them.

### Mobile elements

**Figure 3:**
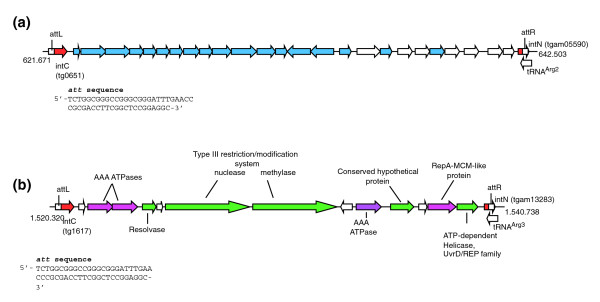
Schematic representation of virus-related loci. **(a) **tgv1 and **(b) **tgv2. Genes are indicated by arrows. Exclusive *T. gammatolerans *genes are not colored. Coordinates are in nucleotides. The respective *att *sequences of each locus are specified. CDS homologues found in *T. kodakaraensis *tkv2 and/r tkv3 virus-like loci [13] are colored in blue (a). CDSs more frequently found in Bacteria than in Archaea are colored in green (b). CDSs well distributed in Archaea are colored in purple (b).

An important feature of the *T. gammatolerans *genome is the absence of genes encoding transposases found in other Archaea, indicating they have not played a role in the evolution of the *Thermococcus *genomes. The genome of *T. gammatolerans *contains two virus-related regions, tgv1 (20,832 bp) and tgv2 (20,418 bp) (Figure 3). Both resulted from the integration in the chromosome of a virus or a virus-related plasmid by a mechanism comparable to that proposed for pSSVx/pRN genetic elements found in *Sulfolobus *species [43]. Both site-specific integrations occurred in a tRNA^Arg ^gene and resulted in the partitioning of the integrase gene (*int*) into two domains, each containing the downstream half of the tRNA gene, which overlaps the 5' (intN) and the 3' (intC) regions. These overlapped regions (48 bp) are predicted to contain attachment (*att*) sites of the integrase. A perfect match between intN and intC was revealed in both cases, indicating a recent integration event. The first virus-related region encoded by the locus starting at the gene tg0651 and ending at the open reading frame (ORF) tgam05590 is closely related to the TKV2 and TKV3 genetic elements found in *T. kodakaraensis *KOD1 [13] and to another element present in *P. horikoshii *[10]. The respective amino- and carboxy-terminal domains of the integrases are well conserved within these three species, indicating close homology between these mobile elements. Most of the genes found in these loci encode conserved hypothetical proteins. Those found over the 5' half of the genetic element appear to be more conserved than those spanning the 3' half (Figure 3a). Several CDSs found in the 3' half of TKV2 and TKV3, as well as in *P. horikoshii*, are missing in tgv1. Consequently, among the genes with a functional assignment in *T. kodakaraensis *KOD1, only those coding for a predicted AAA-ATPase (tg0662) [44] and the putative transcriptional regulator (tg0667) are conserved in *T. gammatolerans*. Only three proteins of tgv1 were found in our proteome survey (Tg0665 to Tg0667), indicating a limited contribution of this virus-related region to cell physiology in the culture conditions used in this study.

Interestingly, the second virus-related region, tgv2, encoded by the locus tg1617-tgam13283, as shown in Figure 3b, is unusual in Archaea. In this case, the intN and intC integrase domains have largely diverged from the tgv1/TKV2/TKV3 respective domains, suggesting a phylogenetic difference. Moreover, 8 out of the 14 genes found in tgv2 are predicted to encode proteins of known function: 3 AAA-ATPase proteins (tg1619, tg1620, tg1626), a resolvase (tg1621), a nuclease (tg1623) and a methylase (tg1624) of a type III restriction/modification system, a putative ATP-dependent helicase belonging to the UvrD/REP family (IPR000212, tg1630), and a protein (tg1629) that shares homology (24% identity, 44% similarity) with RepA/MCM proteins encoded in plasmids isolated from *Sulfolobus neozealandicus *[45]. Several of these proteins (Tg1621, Tg1623, Tg1624, Tg1627, Tg1630) are more frequently found in bacteria than in archaea, Tg1619, Tg1620, Tg1626 being well distributed in archaea, whereas Tg1618, Tg1622, Tg1625, Tg1628 have been exclusively found in *T. gammatolerans *so far. Altogether, these results suggest that tgv2 is a new type of virus-related plasmid integrated into the *T. gammatolerans *genome. Both type III restriction/modification system proteins and the conserved hypothetical protein Tg1627 were expressed in the cells at a sufficient level to be detected in our proteome analysis.

### COG functional group distribution of the experimental proteome

Table 2 shows the distribution of proteins identified by mass spectrometry among all predicted functional cluster of orthologous groups (COG) categories. Out of the 1,101 proteins listed in our mass spectrometry proteome analysis (less stringent parameters), 795 (72%) are conserved in all Thermococcales and 915 (83%) are common to the three *Thermococcus *species. These proteins should represent the core Thermococci proteome - that is, a set of expressed ancestral traits - as proposed by Callister *et al*. [46]. While an additional set of 253 proteins is conserved in at least another *Thermococcus *species, 53 proteins are specific to *T. gammatolerans*.

**Table 2:** COG distribution of the *T. gammatolerans *proteome

COG category	Total number	MS-proof number	Total percentage	MS-proof percentage	MS-proof in category percentage
A: RNA processing and modification	1	1	0.05	0.05	100
T: Signal transduction mechanisms	18	15	0.83	0.7	83.33
J: Translation, ribosomal structure and biogenesis	163	119	7.56	5.52	73.01
F: Nucleotide transport and metabolism	54	38	2.5	1.76	70.37
C: Energy production and conversion	129	90	5.98	4.17	69.77
D: Cell cycle control, cell division, chromosome partitioning	19	13	0.88	0.6	68.42
E: Amino acid transport and metabolism	115	78	5.33	3.62	67.83
O: Posttranslational modification, protein turnover, chaperones	62	42	2.87	1.95	67.74
N: Cell motility	18	12	0.83	0.56	66.67
B: Chromatin structure and dynamics	3	2	0.14	0.09	66.67
H: Coenzyme transport and metabolism	65	43	3.01	1.99	66.15
I: Lipid transport and metabolism	23	14	1.07	0.65	60.87
L: Replication, recombination and repair	63	38	2.92	1.76	60.32
K: Transcription	98	59	4.54	2.74	60.2
Q: Secondary metabolite biosynthesis, transport and catabolism	14	8	0.65	0.37	57.14
G: Carbohydrate transport and metabolism	92	52	4.27	2.41	56.52
R: General function prediction only	289	150	13.4	6.95	51.9
M: Cell wall/membrane/envelope biogenesis	41	20	1.9	0.93	48.78
S: Function unknown	185	88	8.58	4.08	47.57
U: Intracellular trafficking, secretion, and vesicular transport	15	7	0.7	0.32	46.67
V: Defense mechanisms	21	8	0.97	0.37	38.1
P: Inorganic ion transport and metabolism	81	26	3.76	1.21	32.1
No COGs	588	178	27.26	8.25	30.27

Genes assigned to three COG categories are under-represented, with less than 40% of those detected falling into the 'no COGs', 'inorganic ion transport and metabolism', and 'defense mechanisms' categories. Such distribution may be due to the growth conditions and/or the specific biochemical properties of the proteins encoded by genes belonging to these COG categories. Surprisingly, 83% of the genes of the 'signal transduction mechanisms' category, including several encoding predicted Ser/Thr protein kinases, as well as genes assigned to metallophosphoresterases and various AAA proteins, were detected. This indicates that proteins belonging to this category are probably necessary whatever the growth conditions. In contrast with this observation, only a very restricted set of phosphorylated peptides were detected (data not shown). Further experiments are needed to examine the post-translational modifications of these proteins more closely. Among the 587 *T. gammatolerans *genes that code for conserved hypothetical proteins and the 135 CDSs that specify orphans, 221 (38%) and 29 (22%), respectively, were definitively validated by mass spectrometry. Interestingly, from the subset of 214 conserved hypothetical proteins found in all Thermococcales species, 120 were detected in our proteome analysis, demonstrating that they are expressed in classic culture conditions. In all these organisms they probably play important roles that remain to be discovered.

**Figure 4:**
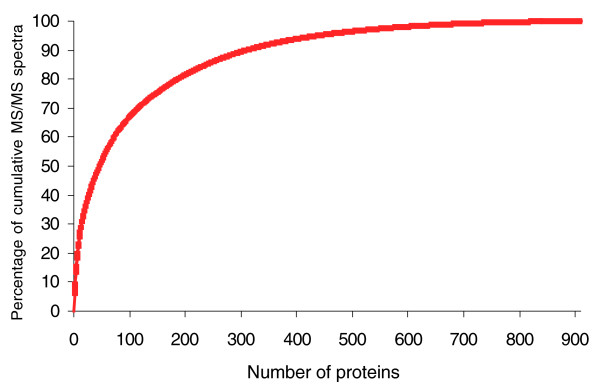
Distribution of protein abundances. The average number of MS/MS spectra was calculated for each protein from two normalized shotgun experiments done on cells harvested in the exponential phase (Table S8 in Additional data file 2). Normalization was done on total MS/MS spectra. The proteins were ranked as a function of their average number of MS/MS spectra from the most to the least detected. The graph reports the percentage of cumulative MS/MS spectra per number of proteins considered.

A biological duplicated analysis was carried out on the proteome content of cells collected in the exponential phase and compared to that of cells harvested during the stationary phase. Spectral counting (Table S8 in Additional data file 2) enables the proteins to be classified in terms of detection level. On this basis, Tg0331, a putative solute binding protein located on the border of a gene cluster identified as a dipeptide ABC-transport system, seems the most abundant protein. After taking into account the molecular weight of the polypeptides, the putative glutamate dehydrogenases Tg1822, Tg1823, and Tg0331 may be considered the three most abundant proteins whatever the growth phase. Interestingly, the conserved protein Tg2082, whose function could not be predicted, is remarkable as it is amongst the 30 most detected proteins. Figure 4 shows the cumulative number of MS/MS spectra recorded against the number of proteins considered, but ranked from the most to the least abundant. These data indicate that, in the exponential phase, only 46 proteins contributed to half of the total number of MS/MS spectra recorded, while 147 and 437 proteins contributed to 75% and 95% of these spectra, respectively.

### Growth requirements of *T. gammatolerans *EJ3

In contrast to what was previously described [20], *T. gammatolerans *EJ3 is able to grow not only on complex organic compounds in the presence of S° but also on a mixture of 20 amino acids or with sugars as carbon sources (Table 3). In the latter case, cells do not require S° but, unlike *P. furiosus *[47], *T. gammatolerans *is obviously not able as to grow on peptides or amino acids without S°. We checked experimentally that *T. gammatolerans *effectively grows like *P. furiosus *and *T. kodakaraensis *KOD1 on complex media that contains starch or maltodextrins as the main carbon source. Similarly, growth using complex media containing pyruvate does not require S° and, like in other Thermococcales species, probably leads to the production of hydrogen instead of hydrogen sulfide when S° acts as final electron acceptor. In a medium supplemented with peptides and S°, the generation time of *T. gammatolerans *cells is 90 minutes and the stationary phase is reached at a cellular density of 5 × 10^8 ^to 10^9 ^cells/ml. The generation time is longer when cells grow on amino acids (4 h in artificial seawater (ASW)-AA) or with sugars (5 h in MAYT-P) and the cellular density is lower (1 to 2 × 10^8 ^cells/ml) than with peptides and S°, indicating a preferential use of peptides and S° for energy and synthesis.

**Table 3:** Carbon sources and S° requirements of *T. gammatolerans *EJ3

		Growth	
		
Carbon source	Media	Without S°	With S°
Yeast extract and tryptone	VSM, MAYT	-	+++
20 amino acids	ASW-AA	-	++
Casamino acids	ASW-CASA	-	-
Yeast extract	ASW-YE	-	+++
Tryptone	ASW-T	-	+++
Peptone	ASW-P	-	+++
Pyruvate	ASW-AA-Pyr	++	++
Pyruvate	MAYT-P	+++	+++
Starch	MAYT-S	+	+++
Maltodextrins	MAYT-Mdx	+	+++
Maltose	MAYT-M	-	+++
Trehalose	MAYT-T	-	+++
Glucose	MAYT-G	-	+++
Lactose	MAYT-L	-	+++

Serum bottles were inoculated at a final concentration of 5 × 10^5 ^cells/ml and incubated at 85°C. Growth was recorded during 3 days. All tests were performed in triplicate. Final cellular density reached at the stationary phase: +++, >5 × 10^8 ^to 10^9 ^cells/ml of culture; ++, 1 to 2 × 10^8 ^cells/ml of culture; +, 5 × 10^7 ^cells/ml of culture; -, no growth.

Amino acid auxotrophy assays show that *T. gammatolerans *does not require for growth any of the 12 following amino acids: Ala, Asn, Asp, Glu, Gln, Gly, His, Ile, Pro, Ser, Thr and Tyr (Additional data file 4). In accordance with auxotrophic requirements, *T. gammatolerans *is able to grow on plate on minimal ASW medium supplemented with nine essential amino acids: Cys, Leu, Lys, Met, Phe, Trp, Val, Arg and Thr and S°. In this case, one of these amino acids, such as Thr, had to be added to the growth medium in a larger amount to be used as carbon source. Casamino acids produced by acid treatment lack Trp, Asn and Gln and, therefore, cannot be used as sole carbon source for growth in minimal ASW medium (Table 3).

### *T. gammatolerans *EJ3 general catabolism as determined by inspection of its genome and proteome

**Figure 5:**
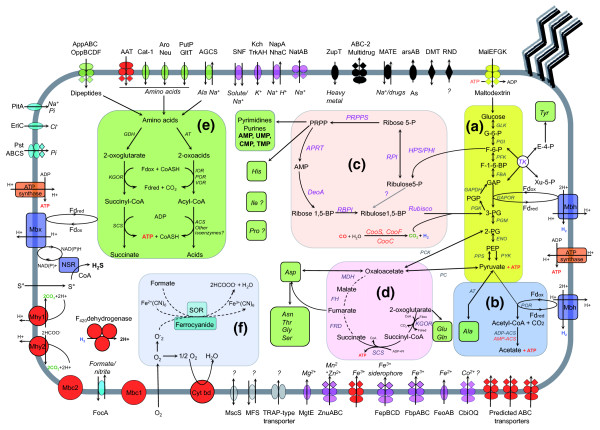
Predicted general metabolism and solute transport in *T. gammatolerans*. **(a) **Modified Embden-Meyerhof glycolytic pathway. **(b) **Pyruvate degradation. **(c) **Pentose phosphate synthesis and carbon dioxide fixation. **(d) **Pseudo tricarboxylic acid cycle. **(e) **Amino acid degradation. **(f) **Oxygen and reactive oxygen species detoxication. The transporters and permeases deduced from the annotatable CDSs are grouped by substrate specificity: anions (blue), amino acids/dipeptides (green), cations (pink), heavy metal or drug (black), carbohydrates (yellow) and unknown (grey). Dashed lines represent pathways not yet experimentally validated in Thermococcales species. Red illustrates proteins only found in *T. gammatolerans *or shared with *T. onnurineus *(Mhy1, Mhy2 and F_420 _dehydrogenase). A detailed legend of Figure 5, including gene ID, is available in Additional data file 5.

We present here a predicted general metabolism of *T. gammatolerans *based on the high level identity of proteins (Figure 1a and Table 1) involved in pathways already experimentally validated in other Thermococcales species (Figure 5; Additional data file 5). Furthermore, we assume that these pathways are active under our physiological growth conditions (VSM medium with S°) since we detected the presence of a large majority of these proteins in our proteomic studies. However, *T. gammatolerans *also contains specific features that are discussed below.

In order to assimilate the proteinous substrates, the *T. gammatolerans *EJ3 genome encodes a putative extracellular archaeal serine protease (tg2111), a pyrolysin homologue (tg1044) [48] and a subtilisin-like protease (tg0368). Unlike in the *T. kodakaraensis *KOD1 genome, no thiol protease gene could be localized. Peptides generated by such proteases might be imported through ABC-type transporters of the dpp/opp family. Such a transporter (tg0383-385) is only found in *T. gammatolerans*. The peptides would be further digested by the numerous predicted proteins with proteolytic or peptidolytic activities (leucine and methionine aminopeptidases, carboxypeptidases, endopeptidases, dipeptidases). Amino acid transporters (tg0308, tg0963, tg1060, tg1321, tg1756, and tg1855) ensure that *T. gammatolerans *can grow using amino acids as the sole carbon source in the presence of S° (or Cys). Among them, genes (tg0091, tg0092, tg0094, tg0095) belonging to the Polar amino aid uptake transporter (PAAT) family, putatively involved in glutamine transport, are only found in the Archaea in *T. gammatolerans*.

According to the amino acid auxotrophies mentioned above, genes coding for proteins of the biosynthetic pathways of ten amino acids (Ala, Asn, Asp, Glu, Gln, Gly, His, Ser, Thr and Tyr) were identified (Additional data file 4). Genes involved in His (tg1607 to tg1614) and Tyr (tg1589 to tg1598) biosynthesis pathways were found clustered as in the other Thermococcales. Like *T. kodakaraensis*, genes involved in Ile, Pro, Arg, Leu, Phe and Val biosynthesis are missing in *T. gammatolerans*. However, this species is able to grow without Ile and Pro. Such discrepancy between gene content and auxotrophic requirements may be explained by novel pathways for Ile and Pro biosynthesis that remain to be discovered. In contrast to *T. kodakaraensis*, neither methionine nor cysteine synthases could be predicted in the *T. gammatolerans *genome. This explains the auxotrophy observed for sulfur-containing amino acids. Moreover, the genes involved in the non-conventional prokaryotic Lys biosynthesis pathway through α-aminoadipic acid [49] could not be identified. In addition, only the last enzyme of the Trp biosynthesis pathway (tg1811), tryptophane synthase, was detected by similarity whereas the whole pathway is encoded by clustered genes in *T. kodakaraensis*. Even if the cells grew in a rich medium, we observed with the shotgun proteomics approach most of the enzymes involved in the biosynthesis pathways of the ten non-essential amino acids. This is somewhat surprising as numerous ABC amino acid transporters are also found, and suggests that the cells maintained a subtle compromise between import and biosynthesis of these compounds.

The amino acids extracted from peptides or imported by transporters are metabolized by transaminases and four distinct ferredoxin oxidoreductases (pyruvate:ferredoxin oxidoreductase (pyruvate:ferredoxin oxidoreductase, 2-oxoisovalerate:ferredoxin oxidoreductase, indolepyruvate:ferredoxin oxidoreductase, 2-ketoglutarate:ferredoxin oxidoreductase) into their corresponding CoA derivatives [50]. Deamination occurs in a glutamate dehydrogenase-coupled manner that differs from other Thermococcales species; this is because in *T. gammatolerans *glutamate dehydrogenase is probably not monomeric as we identified by similarity two genes (tg1822, tg1823) corresponding to a split glutamate dehydrogenase, a situation reminiscent of that found with *Methanosarcina mazei *genes MM3297 and MM3298. These compounds are then further transformed into the corresponding acids by acetyl-CoA synthetases and succinyl-CoA synthetases, respectively [13,51]. This final step, consisting of the conversion of acetyl-CoA/succinyl-CoA into acids, produces energy through concomitant ADP phosphorylation. A unique feature of *T. gammatolerans *among Thermococcales species is the presence of an acetate CoA ligase (EC 6.2.1.1; tg0230) that may produce ATP and acetate from acetyl-CoA and CO_2 _or, conversely, could transform acetate into acetyl-CoA accompanied by AMP formation. Interestingly, this protein is among the most abundant found in *T. gammatolerans *as judged by the spectral counting recorded in our proteome analysis. Why this protein is so abundant remains to be determined. Alternatively, *T. gammatolerans *may also metabolize 2-oxoacids (Figure 5) through ferredoxin oxidoreductases into the corresponding aldehydes as proposed by Ma *et al*. [52]. Aldehydes would be subsequently oxidized by a tungsten-containing aldehyde:ferredoxin oxidoreductases (candidates genes include tg1913 and tg1732) or transformed into alcohol by alcohol dehydrogenase (a candidate gene being tg1572). In order to explore the proteome of this organism, cells were cultivated in a rich-medium containing peptides and S°. Consequently, all the proteins assigned to these different pathways are found.

As mentioned before, *T. gammatolerans *can also use different sugars to grow (Table 3); S° is not required in this case. Since *T. gammatolerans *encodes extracellular α-amylase (tg0222) [53], pullulanase (tg1752) and several putative amylopullulanases (tg0603, tg0690, tg1390), this strain may be able to cleave α-1,4 and α-1,6 bonds between glucose units found in starch or maltodextrins. In contrast to *P. abyssi *and *P. horikoshii*, we could not identify in the *T. gammatolerans *genome any candidate involved in β-glucan degradation [10,12]. Maltooligosaccharides produced by α-glucan degradation could be imported by a MalEFGK transporter (encoded by genes tg0600, tg0601, tg0602, and tg0604; Figure 5) as observed in *P. furiosus *[54]. *T. gammatolerans *does not grow on maltose or trehalose (Table 3), which is consistent with the absence of the α-glucoside ABC-transport system, which is dedicated to maltose or trehalose uptake in *P. furiosus *[54]. Imported oligosaccharides are then reduced into monosaccharides by intracellular α-glucanotransferase (preferentially Tg1711 rather than Tg2132, as indicated by our spectral count approach) [55], maltodextrin phosphorylase (tg1772) and α-glucosidase (tg1709) [56]. Interestingly, among the proteins identified by our shotgun proteomics approach, all these proteins (except tg0222 and tg0602) were synthesized despite sugar or pyruvate being omitted from the culture medium. We assume they are constitutively expressed or, alternatively, are induced either by sugars secreted by the cells or by traces found in peptone and/or the yeast extract.

Among the proteins identified by our shotgun proteomics approach, we found most of the enzymes involved in the archaeal modified Embdem-Meyerhof pathway [57] and the non-oxidative pentose phosphate pathway [58]. Moreover, as recently described in *T. kodakaraensis *[59], the CO_2 _formed by catabolism could be a substrate for the RuBisCo enzyme (Tg1751), which together with the AMP phosphorylase (Tg1786) and the ribose-1,5 biphosphate isomerase (Tg1633) produces 3-phosphoglycerate, thus activating the glycolysis/neoglucogenesis pathways. These proteins are abundant in the cells, as revealed by our proteome analysis.

### Hydrogenases and other related membrane-bound complexes

**Figure 6:**
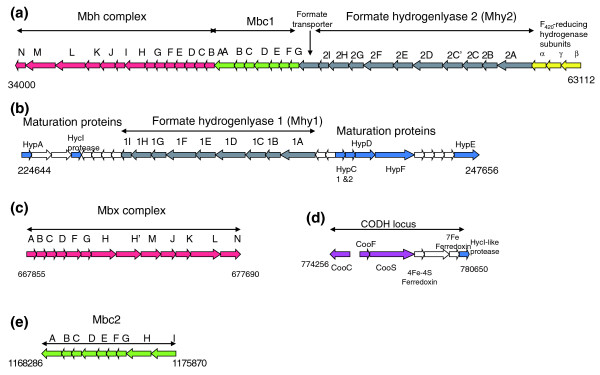
Schematic representation of the loci encoding five membrane-bound complexes. Genes are indicated by arrows. Coordinates are in nucleotides. The genes are colored according to their structural homology (formate hydrogenlyase 1 and 2 in grey; Mbh/Mbx in red, Mbc1 and Mbc2 in green, CODH in purple, maturation proteins in blue). Exclusive *T. gammatolerans *genes are not colored.

A large variety of hydrogenase complexes exist in *T. gammatolerans *but their nature and composition differ to those found in *T. onnurineus *and also in other Thermococcales. They are grouped within five clusters of genes (Figure 6). Like in all other sequenced Thermococcales species, *T. gammatolerans *encodes orthologues of the membrane-bound hydrogenase (Mbh) and membrane-bound oxidoreductase (Mbx) complexes [60] (Table S12 in Additional data file 6). The H_2_-evolving Mbh pumps protons across the membrane and the resulting proton gradient is used by ATP synthase to form ATP. Although experimental evidence is lacking, it was proposed that the Mbx complex reduces ferredoxins and NAD(P), the latter being used by the NAD(P)H elemental sulfur oxidoreductase (NSR) to reduce S° into H_2_S [61]. Since most of the Mbx subunits (12 out of 13) and the NSR orthologue (tg1050) are found in our proteome, we assumed that *T. gammatolerans *likely reduces S° into H_2_S this way. Almost all Mbh subunits (9 out of 14) have also been identified in the proteomic analysis. Based on our semi-quantitative estimation of the amount of proteins in cells harvested during the exponential phase, Mbx components seem to be more abundant than Mbh subunits (appearing in 717 MS/MS spectra versus 83 MS/MS spectra, respectively). The same ratio was found in cells harvested during the stationary phase. These results indicate that Mbx is probably preferentially used in the culture conditions used for this proteomic study, in agreement with previous observations made with *P. furiosus *[61]. In this archaeon, the genes encoding Mbh subunits were found to be rapidly down-regulated in the presence of S° [61], impairing H_2 _production. After data normalization to take into account the size of each polypeptide, we noted in our spectral count that the J, K, and L subunits are the most detected of the Mbx subunits. We observed a drastic change in Mbx subunit ratio when the cells changed from the exponential to the stationary phase. While the F, G, H, H' and M subunits are abundant in the former phase, they are not detected at all in the latter. Williams *et al*. [62] showed that Mbx and Mbh genes of *P. furiosus *grown with maltose are differentially expressed after irradiation. The Mbh complex is down-regulated whereas Mbx is up-regulated. Such anti-correlated regulation may be necessary to re-adapt metabolism quickly, a trait probably common to all Thermococcales species.

As shown in Figure 6, several other membrane-bound complexes containing numerous proteins homologous to Mbh subunits were identified on a sequence similarity basis and in a genome context. We propose the existence of at least two new membrane-bound complexes, which we name here membrane-bound complex 1 (Mbc1) and Mbc2 (Table S13 in Additional data file 6). Mbc1 (tg0048 to tg0054) is probably anchored at the membrane because of the presence of Mbh A-D and MbhF-G-like proteins. A Mbh H-like subunit, related to the NADH dehydrogenase NuoL from many organisms and the HyfB subunit of hydrogenase 4 from *Escherichia coli*, was found. Mbc2 (tg1241 to tg1249) probably comprises six equivalent anchor subunits together with three other proteins that, like in Mbc1, are encoded in the close neighborhood of the membrane subunits. None of these proteins contains the conserved cysteine motifs chelating Fe or Ni that are usually found in other hydrogenase or oxidoreductase subunits. We detected in the shotgun proteomics analysis only two subunits composing the Mbc1 complex and no Mbc2 components. These two complexes appeared to be much less abundant than Mbh and Mbx in the culture conditions used.

A second locus encodes a formate hydrogenlyase (Mhy1) closely related to that recently described in *T. litoralis *[63] (Table S14 in Additional data file 6). It is composed of a formate dehydrogenase and a type-3 hydrogenase. Another putative hydrogenlyase (Mhy2) is encoded by the cluster tg0056 to tg0065 (Table S15 in Additional data file 6). It is composed of a formate dehydrogenase and a new hydrogenase. As shown in Figure 6, both hydrogenlyases share almost the same subunits. A subunit involved in proton translocation is duplicated (Mhy2C and Mhy2C'), resulting in the presence of three such homologous subunits taking into account Mhy2D. This feature resembles that of bacterial type-4 hydrogenase, which also contains three orthologous subunits (HyfB, HyfD, and HyfF). Likewise, a gene coding for a formate transporter gene (FocA; tg0055) is found at the 3' end of the Mhy2 cluster, as in *E. coli*. In the culture conditions used, no subunits of hydrogenlyase II were detected. This result is comparable to what was reported from the proteome analysis of *T. onnurineus *[8] and for *T. litoralis*, where the genes encoding hydrogenlyase I were found to be down-regulated when supplying peptides with S° [63]. Interestingly, by comparative genomics of Mhy loci, we discovered additional conserved genes (tg0056, tg0241, TON_0273, TON_1572, PAB1398.2n) located at the 3' end of the operons in *T. gammatolerans*, *T. litoralis *and *P. abyssi*. As these genes encode a short polypeptide (that we named MhyI), they were not considered during *T. litoralis *formate hydrogenlyase I annotation [63]. A comparison of MhyI sequences shows that the proteins have diverged even if several amino acids are conserved (Additional data file 7). We propose that these proteins may be additional hydrogenlyase subunits or play a role in regulation of these complexes. Several copies of hydrogenases were also found in *T. onnurineus *[8] but their distribution in the genome as well as their complexity in terms of subunit content differ between both species. As an example, the *T. onnureinus *hyg4-1 locus encodes a homologue of Mhy1 clustered with some subunits of the Mbc2 complex. Further analyses will be necessary to elucidate their respective roles in cell physiology.

The origin of formate remains obscure in archaea. In contrast to *T. kodakaraensis*, *T. gammatolerans *does not encode a pyruvate formatelyase able to produce formate and acetyl-CoA from pyruvate [13]. In the other Thermococcales species, anaerobic peptide fermentation likely produces formate through an as-yet uncharacterized pathway. Kletzin and Adams [64] showed that aldehyde:ferredoxin oxidoreductase is able *in vitro *to use formaldehyde as substrate to produce formate. This protein, encoded in *T. gammatolerans *by tg0122, was detected in our proteomic studies. Another hypothesis already suggested for two methanogenic archaea is that an association of formate dehydrogenase with the FAD_420_-reducing dehydrogenase produces formate by the reduction of CO_2 _with hydrogen [65,66]. In this light, the efficiency of several metabolic pathways in *T. gammatolerans *could be directed by the cellular concentration of H_2_, formate and F_420_.

Surprisingly, no gene encoding soluble heterotetrameric NiFe-hydrogenase was detected by sequence similarity in the *T. gammatolerans *genome while one or two (Hyh1 and Hyh2) are found in all hitherto sequenced Thermococcales (Table S12 in Additional data file 6). Their respective roles are unclear. It was proposed that they could serve to recycle H_2 _and reduce NADPH for biosynthesis [60,67]. The tg0066 to tg0068 locus specifies a heterotrimeric reducing F_420_H_2 _hydrogenase. This enzyme is found only in *T. onnurineus *and archaeal methanogens, where it is involved in carbon dioxide reduction via the methane metabolic pathway (reviewed in [68]). The intracellular pool of reduced F_420 _in methane-forming cells is in equilibrium with the H_2 _concentration in the medium [69]. Therefore, F_420_-linked processes would be directly coupled to H_2 _levels. Further experiments will be necessary to conclude if this enzyme compensates for the absence of Hyh1 and Hyh2 in *T. gammatolerans*.

Like in *T. onnurineus*, *T. gammatolerans *has putative CO dehydrogenase (CODH) genes (*CooC*, *CooF*, *CooS*), suggesting that this organism is also able to oxidize CO into CO_2_. Such activity was previously described in *Thermococcus *sp. strain AM4, which is phylogenetically close to *T. gammatolerans *[9], but the genes were not characterized. Lee *et al*. [8] failed to detect the genes by PCR in seven other *Thermococcus *species. Consequently, we report here evidence of CODH in a second *Thermococcus *species. Interestingly the CooC and CooS proteins are present in cells even if they grow in a rich medium supplied with S°, while *CooS *was strongly down-regulated in *T. onnurineus *and no CODH subunits were detected [8]. In this species, the CODH genes are encompassed in a large cluster (TON_1016 to TON_1031) containing a transcriptional regulator and homologous subunits of both Mbc2 and hydrogenlyases. As shown in Figure 6, this is not the case in *T. gammatolerans*. Consequently, the regulation and the role of CODH may differ in both species.

### Hydrogenase maturation systems

The *T. gammatolerans *genome encodes homologues of the known bacterial proteins HypA, HypC, HypD, HypE, HypF, and HycI, which are involved in the insertion of the heterodinuclear center into [Ni-Fe] hydrogenases. The corresponding genes are in the vicinity of the Mhy1 locus (Table S16 in Additional data file 6; Figure 6). Of note, the HypB GTPase essential for nickel insertion in conjunction with HypA in Bacteria (reviewed in [68]) is missing in all Thermococcales species. Hydrogenase activity can be partly restored in a *hypB E. coli *mutant by supplying high concentrations of Ni^2+ ^into the medium [70]. As the sequenced *Thermococcus *species have been isolated from various deep-sea vents rich in various metal elements, the absence of a gene encoding a HypB-like protein may be a consequence of the presence of sufficient nickel concentrations in the *Thermococcus *biotope. Finally, genome analyses also show the presence of two genes encoding HypC-like proteins in *T. gammatolerans *and two copies of a gene encoding a HypD-like protein in *T. onnurineus*, which probably results in structural differences in the hydrogenase complexes of these two species.

### Detoxification systems

The *T*. *gammatolerans *genome includes genes for several detoxification enzymes. By analyzing sequence similarities, we could assign a thioredoxin reductase (tg0180), a glutaredoxin-like protein (tg1302) and two peroxiredoxins (tg1253, tg1220), which could allow the archaeon to cope with oxidative stress. In order to eliminate the superoxide ions, several mechanisms have been proposed for archaea. One mechanism involves ferrocyanides, formate and the superoxide reductase [71]. Several transporters of iron and formate are present, as well as the superoxide reductase enzyme. Another alternative pathway has been described in which the superoxide reductase is associated with rubredoxin and rubrerythrin [72], but no gene encoding rubredoxin could be identified in *T. gammatolerans*. Interestingly, this organism harbors genes (tg1232 to tg1233) encoding proteins similar to cytochrome bd ubiquinol oxidase and homologous to the bacterial CydAB [73]. This enzyme functions as a quinol oxidase and protects anaerobic processes from inhibition by oxygen [74]. To our knowledge, we report here the first description of these genes in a Thermococcales species. Moreover, the genes are located in the close neighborhood of a putative operon coding for several proteins described as involved in the ubiquinone synthesis pathway (Kyoto Encyclopedia of Genes and Genomes (KEGG) pathway ko00130): a methyltransferase belonging to the ubiE/COQ5 family (EC:2.1.1.-; Tg1226), 3-octaprenyl-4-hydroxybenzoate carboxy-lyase (EC:4.1.1.-; Tg1231) and 4-hydroxybenzoate polyprenyltransferase (EC 2.5.1.-; Tg1230). These proteins are homologous to counterparts found only in thermophilic bacteria such as *Geobacter sulfurreducens PCA*, *Desulfovibrio desulfuricans *G20, *Symbiobacterium thermophilum*, *Moorella thermoacetica ATCC 39073 *and *Aquifex aeolicus*. The genes have probably been acquired by horizontal gene transfer.

### Known DNA repair arsenal in *T. gammatolerans*

Since *T. gammatolerans *exhibits a radioresistance still unequalled in archaea, and to draw a consistent picture of the DNA repair arsenal of this organism, we searched for the presence of genes involved in DNA replication, repair or recombination described in other archaea, or detected through sequence similarities, specific motifs and domains in public databases.

The proteins involved in DNA replication in other archaea (cdc6, RF-C, MCM, primase, polymerases B and D, Fen1 endonuclease, GINS, RPA proteins, helicases, topoisomerases, and so on; reviewed in [75]) are all found in *T. gammatolerans *(Table S1 in Additional data file 1). On the other hand, a distant counterpart of the protein Din2 found in the genome of *P. abyssi *is missing. *T. gammatolerans*, as with other thermophilic archaea, has mechanisms to control the pool of nucleotides, and to correct or modify bases or to delete them, creating abasic sites that can be repaired by a base excision repair pathway. *T. gammatolerans *possesses the genetic information for a nucleoside triphosphate phosphohydrolase (tg0168; EC 3.6.1.15) homologous to *Saccharomyces cerevisiae *Hamp1p [76], an ADP ribose pyrophosphatase (tg1861), a homologue of mutT previously characterized in the archaeon *Methanococcus janashii *[77], a 3-methyladenine DNA glycosylase-related protein (tg1192, IPR004597) and also a homologue of the *T. kodakaraensis *O^6^-methyl guanine methyl transferase (tg0325) [78] that directly corrects lesions on DNA. Putative type III (IPR005759; Tg1277), IV (IPR001719; Tg1446), and V (IPR007581; Tg0915) endonucleases, which act at abasic sites generated by high temperature-induced depurination, several predicted DNA glycosylases (Tg0543, Tg1653, Tg1814, IPR05122) and AP endonucleases (Tg0205, Tg0740, Tg1637, IPR001719) are also found as well as the Kae1 protein (Tg0271), which was recently found to be a new AP-lyase protein acting *in vitro *at apurinic sites [79]. Five homologous proteins involved in the nucleotide excision repair pathway in *Pyrococcus *were identified (reviewed in [80]): Tg1658 (XPB-rad25 helicase homologue), Tg0797 (XPD-rad3 helicase homologue), Tg1167 (XPF nuclease), and Tg1199 (XPG/Fen1-like nuclease). Homologues of archeal proteins that may be involved in *T. gammatolerans *double-stranded DNA break repair through homologous recombination include: Tg0130/RadA protein [81], which may possesses, as in *Sulfolobus *and *Pyrococcus*, a DNA-dependent ATPase activity and catalyze strand exchange *in vitro *[82,83], RadB (Tg2074; a truncated version of RadA that may, as in *Pyrococcus*, regulate homologous recombination proteins [84]), Tg1742 and Tg1743 (homologues of the Rad50-Mre11 archaeal proteins [85]), Tg1741 (homologue of the *Sulfolobus *NurA nuclease) [86,87] and Tg1744 (HerA/Mla-like bipolar helicase) [88,89]. The four last genes are found in many thermophilic archaea and the proteins from *P. furiosus *form, *in vitro*, an initiator complex that generates the single strand extremities necessary for homologous recombination [90]. Moreover, *T. gammatolerans *also encodes homologues of the resolvase Hjc (Tg0717) [91], two ligases (Tg1718, Tg2005) and also several putative nucleases belonging to distinct families: tg0136 and tg1824 code for predicted thermonucleases (IPR006021), Tg0864 is a homologue of the *Methanococcus janashii *recJ-like single-stranded exonuclease [92] and Tg1631 contains a TOPRIM domain found in various type IA and II topoisomerases, DnaG-type primases, OLD family nucleases and RecR proteins [93]. Finally, tg1177 encodes a predicted excinuclease ABC C subunit (IPR000305). A set of genes that may constitute a novel 'thermophile-specific' DNA repair system [94] was found to be induced by gamma irradiation in *P. furiosus *[62]. Further *in silico *analysis showed that these proteins are homologous to CRISPR (clustered regularly interspaced short palindromic repeat)-associated proteins [95]. It could also be the same for tg1298, which encodes a putative nuclease clustered in the genome with one CRISPR locus. Finally, a homologue of Rad55 (ST0579) described in *S. tokodaii *[96] is encoded by tg0280. Here, we report that *T. gammatolerans *encodes six paralogues of tg0280 (tg0108, tg0530, tg0616, tg0617, tg0996, tg1736) that, like RadB, are composed only of a highly conserved ATPase domain with an average size of 230 amino acids. Whether these recA-like proteins are involved in repair pathways has to be investigated, as well as their respective roles.

Several lines of evidence suggest that DNA repair genes may be constitutively expressed in archaea. Constant cellular amounts are probably necessary to maintain genome integrity and cope with environmental stresses [62,97]. Several, but not all, were detected in our protein catalog prepared for non-irradiated cells, suggesting they are constitutively expressed in *T. gammatolerans*. The predicted nucleases Tg0864, Tg1177, Tg1631, both ligases (Tg1718, Tg2005), the endonucleases, AP-lyases and glycosylases Tg0271, Tg0543, Tg1192, Tg1446, Tg1637, Tg1814 (putatively involved in base excision repair), Tg0130, Tg0280, Tg1742, Tg1743, Tg1744 and Tg2074 (presumed to be involved in double-strand DNA break repair) were detected while NurA (Tg1741) was not at a sufficient level to be identified. The absence of nucleotide excision repair proteins in the protein catalog (with the exception of Tg1199) indicates that these proteins are in low amounts in the cells and may be specifically synthesized in some given environmental conditions.

**Figure 7:**
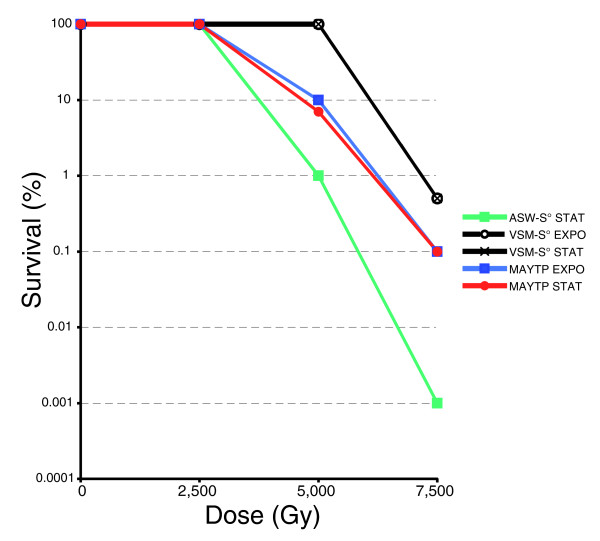
Percentage survival of *T. gammatolerans *after gamma radiation in different media. MAYTP cells were irradiated in the exponential phase (blue squares) or the stationary phase (red circles) under anaerobic conditions. These values are a mean of three independent experiments. Other survival curves were taken from [21].

**Figure 8:**
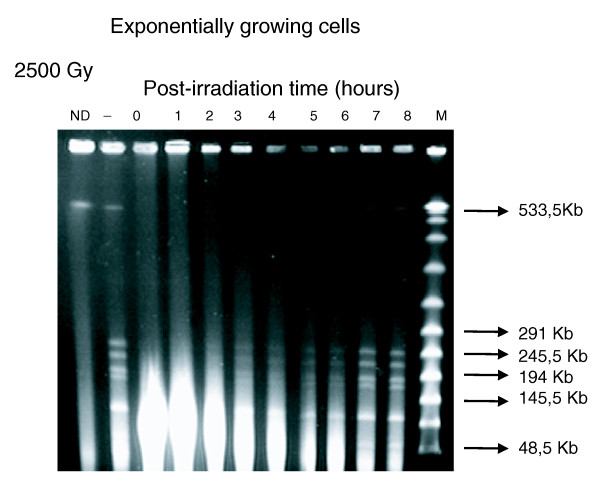
Chromosomal repair kinetics of exponentially growing *T. gammatolerans *cells in MAYTP following irradiation at a dose of 2.5 kGy. Time 0 represents an incubation of 5 minutes at 85°C. Genomic DNA was visualized after digestion by the rare cutting enzyme *Swa*I. The ND lane corresponds to non-digested genomic DNA of non-irradiated cells. The '-' lane corresponds to the digested genomic DNA extracted from non-irradiated cells. A DNA marker (lambda concatamer DNA, PFGE Marker; New England Biolabs, Ipswich, MA, USA) was used as reference. The size of several bands is indicated by arrows.

We investigated the recovery of irradiated *T. gammatolerans *cells in a rich medium containing peptides and S° (VSM-S°) and under nutrient-limiting conditions (20 amino acids supplemented with S° (ASW-S°)) [21]. In the latter case, cells became more sensitive to gamma rays, with a survival of only 0.001% at a dose of 7,500 Gy. In a rich culture medium, *D. radiodurans *and *D. geothermalis *withstand higher doses of irradiation than *T. gammatolerans *[98]. On the other hand, in minimal culture medium, an irradiation dose of 3,000 Gy is lethal for *D. radiodurans *[99]. Exponentially growing *T. gammatolerans *cells exhibit a similar behavior [21]. Since *T. gammatolerans *is also able to grow in the presence of sugar and without S° (MAYTP), we tested here the viability of the cells in such growth conditions. Cell survival in both MAYTP and VSM-S° media are almost the same whatever the phase considered: 100% viability until 2,500 Gy and 0.1% survival at 7,500 Gy (Figure 7). The slight survival increase observed in VSM-S° compared to in MAYTP is probably due to a protective effect of the added sulfur in VSM-S° [100]. We conclude from these observations that the radioresistance of *T. gammatolerans *is not drastically influenced by the metabolic pathways used (peptide degradation versus sugar-based metabolism) nor correlated with the generation time. Generation times in MAYTP and VSM-S° media differ: 5 h and 1.5 h, respectively. Figure 8 shows the kinetics of reconstitution of shattered chromosomes after a 2,500 Gy exposure (100% survival). Whereas 4 h were required for reconstitution when cells were grown in VSM-S° [21], 7 h were needed for cells recovering in MAYTP. As the survival rates are the same in both conditions (100%), we confirmed that the speed of genome restoration after irradiation is not a key parameter for survival, at least in the growth conditions used. In a rich medium, the rates of irradiated DNA recovery appear to be slower than those reported for *D. radiodurans *[101,102]. As shown in Table S17 in Additional data file 8, only a few repair genes in *T. gammatolerans *have homologues in both *Deinococcus *sequenced genomes, but archaeal DNA repair systems, as well as other mechanisms involved in DNA metabolism, are similar to their eukaryotic counterparts and not to those found in bacteria. *D. radiodurans *encodes efficient DNA repair systems but several proteins involved in these pathways remain to be identified [24,102]. Analysis of the transcriptome of *D. radiodurans *revealed a group of genes that are up-regulated in response to either desiccation or ionizing radiation [103]. The five most highly induced genes in response to each stress encode proteins of unknown function and their inactivation indicates that they play roles in radioresistance in recA-dependent and recA-independent processes, but none have homologues in *T. gammatolerans*. However, among hypothetical proteins, one cannot exclude the presence of functional analogues that play comparable roles in the cell. Despite increasing literature on this subject, DNA repair mechanisms in Archaea are less well documented than those found in bacteria. Many proteins, and sometimes entire pathways, are either missing or encoded by genes that remain to be identified [80,104]. In every sequenced genome, approximately 20 to 30% of the genes are orphans or code for conserved proteins with unknown function, which may be specific DNA-interacting proteins still to be characterized.

Finally, as shown throughout the numerous studies of *D. radiodurans*, radioresistance is the result of numerous factors such as a high Mn/Fe ratio, which protects proteins from oxidation, as well as nucleoid condensation limiting diffusion of radiation-generated DNA fragments [105,106]. Therefore, radioresistance is not restricted to DNA repair efficiency [24]. Further genomic studies performed on *T. gammatolerans *will be crucial to discover the different cellular mechanisms responsible for its radiotolerance.

## Conclusions

We report here the *T. gammatolerans *genome sequence and the first archaeal genome-wide proteome investigation performed at the sequence annotation stage. The *T. gammatolerans *genome does not encode any transposase elements but harbors a new virus-related element. This study has allowed us to unravel this archaeon's metabolism under rich medium growth conditions. Even if *T. gammatolerans *is grown with peptides and S°, numerous metabolic pathways appear to be active, including those involved in sugar catabolism and amino acid metabolism. Moreover, this archaeon possesses the common arsenal of DNA repair proteins found in other Thermococcales. *T. gammatolerans *shares 1,606 common genes with *T. kodakaraensis *but the latter is much more sensitive to gamma rays, with a survival rate comparable to those of *Pyrococcus *species (data not shown).We did not detect any duplication, nor additional genes, related to DNA repair. The genomic context of the corresponding genes does not give any clues about the existence of new specific genes that could explain massive DNA repair. We show that *T. gammatolerans *cells repair damage caused by gamma radiation with the same efficiency whatever the culture phase. Moreover, survival is comparable when cells use peptides or sugars while the generation time is longer when cells grow without S°. When cells are irradiated, damage to the Mbh and Mbx complexes and various other transporters could be the most deleterious. A large amount of irradiation may affect not only the ability to internalize substrates necessary to produce energy and repair and recycle cellular damaged compounds, but also to remove reduced cofactors that are in excess. The *T. gammatolerans *genome encodes several membrane-bound complexes (formate hydrogenlyases 1 and 2, Mbc1, Mbc2). In contrast to Mbh and Mbx, most of these proteins are not detected by nano-LC-MS/MS, which is indicative of their relatively low abundance in standard growth conditions. Irradiation may enhance the expression of hydrogenlyases that convert formate into protons and CO_2_. Moreover, the CODH genes, which are expressed even if cells do not grow in a limited-nutrient medium and under a CO atmosphere, may increase the pool of CO_2 _for cell metabolism. The carbon dioxide provides a carbon source for metabolism via the Rubisco, the produced protons being used for ATP synthesis. Finally, one specific feature of *T. gammatolerans *is the presence of numerous systems of cellular detoxification to cope with reactive oxygen species produced by gamma rays. In contrast to other Thermococcales, homologues of the bacterial *cydAB *genes were found and may be specifically synthesized after irradiation. Altogether, these results show that *T. gammatolerans *is a new model of choice for studies of radioresistance in archaea.

## Materials and methods

### Strains, media and growth conditions

*T. gammatolerans *EJ3 was grown in serum bottles under anaerobic conditions at 85°C either in complex organic medium (VSM) supplemented with S° (2 g/l) or in MAYT medium as described in [107] supplemented with 5 g/l pyruvate (MAYTP medium). Growth assays were performed in synthetic media (ASW: artificial sea water) supplemented with a mixture of 20 amino acids [108] or supplemented with specific proteinous substrates (Casamino acids, yeast extract, tryptone and peptone) at a concentration of 5 g/l in the presence or absence of S°. Growth assays were also tested in the nutrient-rich complex medium (MAYT) supplemented with sugar carbon sources (5 g/l) in the presence or absence of S°. VSM is composed of 20 g/l NaCl, 0.25 g/l KCl, 0.05 g/l NaBr, 0.02 g/l boric acid, 0.01 g/l SrCl_2_·6H_2_O, 0.5 g/l trisodium citrate, 3 g/l PIPES (piperazine-1,4-bis(2-ethanesulfonic acid)), 1 g/l yeast extract, 4 g/l Bactotryptone, 5 ml 20% MgSO_4_, 1 ml 5% CaCl_2_, 1 ml 5% KH_2_PO_4_. The pH was adjusted to 6.8 by addition of NaOH. Media were sterilized by autoclaving and transferred into individual serum bottles. Air contained in the bottles was first removed using a vacuum and then replaced by N_2_. To reduce the oxygen dissolved in the medium, Na_2_S·9H_2_O at a 0.1% final concentration was added until the color of resazurin sodium salt (1 mg/l) became clear. Typically, serum bottles were inoculated at a cellular density of 5 × 10^5 ^cells/ml and incubated at 85°C. Growth tests were performed in triplicate. Growth was recorded during 3 days.

### Genome sequencing and assembly

*T. gammatolerans *genome sequencing was initiated by constructing several bacterial artificial chromosome (BAC) libraries (insert sizes of 50 to 75 kb) and two shotgun libraries of different sizes (inserts of 3 and 10 kb) that together achieved a tenfold coverage of the 2 Mb genome, as estimated by pulse field gel electrophoresis. Primary end-sequencing yielded about 32,000 reads that could be assembled in 10 contigs ranging between 35 and 487 kb, which covered more than 90% of the genome. Then, 'gap-closure' and 'finishing' phases were performed with several successive steps of primer walks, totaling 328 synthesized oligonucleotides used for sequencing on 171 distinct templates. Assembly, quality assessments and contig editing were performed with the phred/phrap/consed package [109-111].

### Gene prediction, annotation and comparative genomics

Genome annotation and analysis were performed using a custom genome annotation WEB-based platform [112-114]. The complete sequence and annotation of the genome can be accessed using [GenBank:CP001398]. Semi-automated annotation was performed to identify genes by sequence similarity and coding probability using BLASTP [115] and GLIMMER version 2 [116], taking into account ORFs exceeding 90 nucleotides. Manual annotation was completed within the platform using a wide range of integrated tools. Similarity and motif searches were performed in the following databases using the mentioned tools: GenBank nr, SwissProt version 41.0, and the COG databases COG + KOG (7 eukaryal genomes) using BLASTP; CDD version 2.10 [117], COG version 1.0, KOG version 1.0, Pfam version 11.0, and SMART version 4.0 using rpsBlast; INTERPRO version 12.1, PRINTS version 38.0, PROSITE version 19.10, PFAM version 19.0, PRODOM version 2004.1, SMART version 5.0, TIGRFAMs version 4.2, GO, SSF version 1.65, PIRSF version 2.68, GENE3D version 3.0, and PANTHER version 6.0 using Interproscan v4.2 [118]; transmembrane regions using modhmm version 0.91 [119]; pattern matches in Pfam version 8 using HMMER [120]; and tRNAs were identified using tRNA-scanSE [121].

For genome comparative analysis, homologous genes were defined following pairwise comparisons of BLASTP similarity levels between genomes pairs [122], with threshold values of 80% alignment length for each pair member and 0.3 of maximum bitscore to accept a gene pair as homologous. Additionally, gene pairs having an expected *P*-value = 1e-30 and failing only one of the other criteria were included in the homologous gene list. This procedure avoids discarding gene pairs for which alignment length or percentage bitscore falls just below used thresholds, or in which one gene pair member, but not the other, contains an indel or intein.

Synteny graphs were drawn using homologous gene pair coordinates plotted one against another to give scatter plots of conserved sequences [34,116]. Almost identical scatter plots were obtained using BLASTN of 10 kb chopped genomes or by using MUMmer software [116] (not shown).

The number of genome pair recombination events was calculated as follows: we first defined synteny blocks as any succession of 1, 2,. N homologous genes (as defined above) having an identical organization in both genomes, and then the frequency of synteny block lengths was compiled. The frequency distributions thus obtained were fitted to power-law type distributions whose robustness was estimated by calculating the coefficient of determination (R^2^) with a least squares procedure. Consequently, assuming that the observed distributions were generated by a finite number of random events (recombination hits), the number of recombination events within each genome pair is simply given by summing the total number of synteny blocks.

### Protein extracts, SDS-PAGE, and in-gel proteolysis

*T. gammatolerans *cellular pellets from five replicated cultures in VSM-S° medium were resuspended in a lysis buffer containing 7 M urea, 2 M thiourea, 4% CHAPS, 40 mM DTT, 20 mM spermine, 3 mM TRIS/HCl (pH 7.5), and Complete protease inhibitor cocktail (1 tablet for 10 ml; Roche, Basel, Switzerland). Cells were disrupted by sonication at 4°C and cell debris were then removed by centrifugation. Proteins were eventually concentrated (10×) by trichloroacetic acid precipitation. Proteins (30 and 300 μg) dissolved in LDS sample buffer (Invitrogen, Carlsbad, CA, USA) were resolved by SDS-PAGE with 4 to 12% gradient and 12% NuPAGE (Invitrogen) gels. Gels were stained with Coomassie Safe Blue stain (Invitrogen) and then each lane excised into 25 regions (approximately 2.5 mm × 10 mm). Each band was treated and proteolyzed with trypsin as described in [123].

### LC-MS/MS analysis

LC-MS/MS experiments were performed on a LTQ-Orbitrap XL hybrid mass spectrometer (ThermoFisher, Waltham, MA, USA) coupled to an UltiMate 3000 LC system (Dionex-LC Packings, Sunnyvale, CA, USA). Peptide mixtures (0.5 to 5 pmol) were loaded and desalted online in a reverse phase precolumn (C18 Pepmap column, LC Packings), and resolved on a nanoscale C18 Pepmap TM capillary column (LC Packings) at a flow rate of 0.3 μl/minute with a gradient of CH_3_CN/0.1% formic acid prior to injection into the ion trap mass spectrometer. Peptides were separated using a 90 minute gradient from 5 to 95% solvent B (0.1% HCOOH/80% CH_3_CN). Solvent A was 0.1% HCOOH/0% CH_3_CN. The full-scan mass spectra were measured from m/z 300 to 1,700 with the LTQ-orbitrap XL mass spectrometer operated in the data-dependent mode using the TOP7 strategy. In brief, a scan cycle was initiated with a full scan of high mass accuracy in the orbitrap, which was followed by MS/MS scans in the linear ion trap on the seven most abundant precursor ions with dynamic exclusion of previously selected ions.

### Database mining and mass spectrometry data deposition

Using the MASCOT search engine (version 2.2.04), we searched all MS/MS spectra against two protein sequence databases, TGAM_ORF0 and TGAM_CDS1. TGAM_ORF0 is the compilation of sequences produced by translating the longest possible ORFs bordered by a start and a stop codon as defined by the bacterial and plant plastid genetic code [124], and having at least 30 amino acids. It comprises 17,656 polypeptide sequences, totaling 1,634,020 amino acids, with an average of 92 amino acids per polypeptide. The TGAM_CDS1 database is a subset of the TGAM_ORF0 database comprising the 2,157 predicted CDSs, totaling 632,575 amino acids with an average of 293 amino acids per protein. These two databases are accessible at [112]. Searches for trypsic peptides were performed with the following parameters: full-trypsin specificity, a mass tolerance of 5 ppm on the parent ion and 0.7 Da on the MS/MS, static modifications of carboxyamidomethylated Cys (+57.0215), and dynamic modifications of oxidized Met (+15.9949). The maximum number of missed cleavages was set at 2. All peptide matches with a peptide score of at least 25 (average threshold for *P *< 0.001 with the CDS database) were filtered by the IRMa 1.16.0 parser. A false-positive rate of 0.55% was estimated using a decoy database when considering a protein validated with at least one peptide with score above 50. The false-positive rate was 0.00% when considering a protein validated with at least two peptides (very stringent conditions). Further data analyses were performed at an average threshold for *P *< 0.001: semi-trypsin specificity (peptide scores of at least 36), phosphorylated Ser, Thr, and Tyr (+79.9663; peptide scores of at least 31), formylation and acetylation of protein amino termini (peptide scores of at least 31).

Mass spectrometry data were deposited in the PRIDE Proteomics IDEntifications database [125] under accession numbers [PRIDE:#9212 to #9218], and are freely available at [126].

### Semi-relative protein quantification

The number of MS/MS spectra per protein was determined from three independent experiments conducted in similar conditions. The three data sets were normalized with the total number of spectra recorded in each experiment, and compared with the ACFold method described recently [127]. For this, we used the PatternLab software with false discovery rate, fold cut-off, and *P*-value cut-off set at 0.01, 2.0, and 0.01, respectively. The data are presented per class and fold change (Table S8 in Additional data file 2). A total of 843 proteins were detected in these data sets. Among the 270 proteins that satisfied statistical criteria, 80 were found with a low absolute ACFold value, 125 were found to be more abundant in the exponential phase and 65 were more abundant in the stationary phase.

### Cell survival and DNA repair kinetics after gamma irradiation

*T. gammatolerans *cells from MAYTP cultures were incubated on ice, harvested (2,000 g, 20 minutes at 4°C), and resuspended in a limited volume of freshly reduced medium in order to concentrate them tenfold. Equal samples of cells (0.8 ml) were introduced into Hungate tubes, and then irradiated on ice at a rate of 42.5 Gy per minute using a ^137^Cs gamma ray source (IBL637 CisBio International, Institut Curie, Orsay, France). The same number of non-irradiated control cells was incubated on ice without irradiation. Following irradiation, serial tenfold dilutions were prepared in freshly reduced medium until a cellular density of 0.1 cells per tube. One milliliter of each of these dilutions was used to inoculate serum bottles containing 24 ml of fresh medium. These cultures were then incubated at 85°C for a maximum of 9 days. They were checked every 24 h for presence or absence of growth by optical microscopy using a Thoma counting chamber. Cell survival was evaluated according to the last positive dilution where cells were able to restore a high cellular density culture (>10^7 ^cells/ml) by comparison of dilutions of non-irradiated cells used as an internal reference. All dilutions were performed in duplicate and three biological replicates were checked. To follow the DNA-repair kinetics, irradiated and non-irradiated control cells were incubated at 85°C in MAYTP medium at a density of at least 10^7 ^cells/ml. At regular post-irradiation incubation times (each hour), samples were taken to prepare DNA plugs as described in [100] at a cellular density of 10^8 ^cells per plug. Plugs were then washed in 10 mM TRIS/HCl, 1 mM EDTA, pH 8.0 buffer and stored at 4°C in this solution. Just before digestion, plugs were extensively washed in sterile water, incubated 1 h in the buffer of the restriction enzyme supplied by the manufacturer (New England Biolabs, Ipswich, MA, USA) and then digested for 6 h at 30°C with 40 units of *Swa*I enzyme in a volume of 100 μl per plug. The restriction enzyme was inactivated by incubation at 65°C for 20 minutes. Digested chromosomal DNA was analyzed on 1% agarose gels in 89 mM TRIS/Borate, 2 mM EDTA, pH 8.3 buffer using a CHEF-MAPPER electrophoresis system (Bio-Rad, Hercules, CA, USA) under the following conditions: 5.5 V/cm, 10°C, with a linear pulse of 40 s and a switch angle of 120° (-60° to +60°), for 30 h. Pulsed field gel electrophoresis kinetics are representative of at least two independent experiments.

## Abbreviations

ARG: average number of recombinations per gene; ASW: artificial seawater; CDS: coding sequence; CODH: CO dehydrogenase; COG: cluster of orthologous groups; LC: liquid chromatography; Mbc: membrane-bound complex; Mbh: membrane-bound hydrogenase; Mbx: membrane-bound oxidoreductase; Mhy: formate hydrogenlyase; MS/MS: tandem mass spectrometry; ORF: open reading frame; S°: elemental sulfur; tgv: *T. gammatolerans *virus-related locus.

## Authors' contributions

FC conceived and coordinated the study. FC and PF initiated the project. YZ, PW, MD, FC and JW coordinated and conducted genome sequencing. YZ built databases, performed genome assembly, sequence data management, sequence annotation and comparative genomics. JA and PG performed the proteome experiments and the mass spectrometry assignments. JA and FC analyzed the proteomic data. AL and CL determined growth culture requirements. AL contributed to genome analysis and determined auxotrophic requirement. CL performed survival curves and kinetics of reconstitution of shattered chromosomes. FC, JA and YZ assembled and wrote the manuscript.

## Additional data files

The following additional data are available with the online version of this paper: Tables S1 to S5 (Additional data file 1); Tables S6 to S8 (Additional data file 2); Tables S9 and S10 (Additional data file 3); description of the auxotrophic requirement of *T. gammatolerans *deduced from genome analysis and auxotrophic assays (Additional data file 4); a detailed legend to Figure 5, including gene IDs (Additional data file 5); Tables S12 to S16 (Additional data file 6); a figure showing protein sequence alignments of the putative additional MhyI subunits (Additional data file 7); a table listing the *T. gammatolerans *genes conserved in *D. radiodurans *and *Deinococcus geothermalis *(Additional data file 8).

## Supplementary Material

Additional data file 1 All *T. gammatolerans *genes (Table S1); conserved genes among the three sequenced *Thermococcus *species (Table S2); conserved genes in the six sequenced Thermococcales (Table S3); the *T. gammatolerans *genes absent in other Thermococcales (Table S4); and genes present in all sequenced *Thermococcus *species but absent in all *Pyrococcus *species (Table S5). Click here for file

Additional data file 2 All the non-redundant peptides (Table S6) and proteins (Table S7) identified in *T. gammatolerans*; spectral count comparison of proteins detected in exponential and stationary phases per class and fold change (Table S8). Click here for file

Additional data file 3 The 290 amino-terminal peptide signatures identified in *T. gammatolerans *(Table S9) representing 178 non-redundant amino-terminal events (Table S10). Click here for file

Additional data file 4 Auxotrophic requirement of *T. gammatolerans *deduced from genome analysis and auxotrophic assays (Table S11). Click here for file

Additional data file 5 Detailed legend to Figure 5, including gene IDs. Click here for file

Additional data file 6 Subunit content of the soluble heterotetrameric hydrogenases Mbh and Mbx in several *Thermococcale *species (Table S12); *T. gammatolerans *Mbc1 (tg0048 to tg0054) and Mbc2 (tg1241 to tg1249) complex subunits and their respective homologues (Table S13); formate hydrogenlyase 1 subunit content and their respective homologues found in other *Thermococcale *species and in *E. coli *(Table S14); formate hydrogenlyase 2 subunit content and the formate transporter (Foc) (Table S15); and hydrogenase maturation proteins as well as homologous genes found in other sequenced Thermococcale species (Table S16). Click here for file

Additional data file 7 Protein sequence alignments of the putative additional MhyI subunits. Click here for file

Additional data file 8 *T. gammatolerans *genes conserved in *D. radiodurans *and *Deinococcus geothermalis*. Click here for file
