# Supplementary material for: Genome analysis and genome-wide proteomics of Thermococcus gammatolerans, the most radioresistant organism known amongst the Archaea
Source: Genome Biol. 2009 Jun 26;10(6):R70. doi: 10.1186/gb-2009-10-6-r70 (PMC2718504; doi:10.1186/gb-2009-10-6-r70)
Supplement: Additional data file 4 — Auxotrophic requirement of T. gammatolerans deduced from genome analysis and auxotrophic assays (Table S11). [file gb-2009-10-6-r70-S4.doc]

Table S11: Auxotrophic requirement of *T. gammatolerans* deduced from genome analysis and auxotrophic assays. Predicted amino acid biosynthesis pathways from other Thermococcales (*T.* *kodakaraensis, P. furiosus, P. abyssi, P. horikoshii*) are indicated for comparison.

| Amino acid |  | Auxotrophy (A) / Prototrophy (P) |  | Predicted amino acid  biosynthesis pathway | | | | |
| --- | --- | --- | --- | --- | --- | --- | --- | --- |
|  |  |  |  |  | | | | |
|  |  |  |  |  |  |  |  |  |
|  |  | *T. gammatolerans* |  | *T. gammatolerans* | *T. kodakaraensis* | *P. furiosus* | *P. abyssi* | *P. horikoshii* |
|  |  |  |  |  |  |  |  |  |
| Arg |  | A |  | - | - | + | - | - |
| Cys |  | A |  | - | + | + | + | - |
| Leu |  | A |  | - | - | -^§^ | -^§^ | - |
| Lys |  | A |  | - | + | + | + | + |
| Met |  | A |  | - | + | -^&^ | + | + |
| Phe |  | A |  | - | - | + | - | - |
| Trp |  | A |  | - | + | + | + | - |
| Val |  | A |  | - | - | -^§^ | -^§^ | - |
| Ala |  | P |  | +^#^ | +^#^ | +^#^ | +^#^ | +^#^ |
| Asn |  | P |  | + | + | + | + | + |
| Asp |  | P |  | + | + | + | + | + |
| Glu |  | P |  | + | + | + | + | + |
| Gln |  | P |  | + | + | + | + | + |
| Gly |  | P |  | + | + | + | + | + |
| His |  | P |  | + | + | + | - | - |
| Ile |  | P |  | - | - | -^§^ | -^§^ | - |
| Pro |  | P |  | - | - | - | - | - |
| Ser |  | P |  | + | + | + | + | + |
| Thr |  | P |  | + | + | + | + | + |
| Tyr |  | P |  | + | + | + | - | - |
|  |  |  |  |  |  |  |  |  |

§: only the last enzyme of this biosynthesis pathway lack in these strains

&: only partially predicted pathway

#: an alanine aminotransferase (aat) could be predicted from these genomes
