# Supplementary material for: Genome analysis and genome-wide proteomics of Thermococcus gammatolerans, the most radioresistant organism known amongst the Archaea
Source: Genome Biol. 2009 Jun 26;10(6):R70. doi: 10.1186/gb-2009-10-6-r70 (PMC2718504; doi:10.1186/gb-2009-10-6-r70)
Supplement: Additional data file 5 — Detailed legend to Figure 5, including gene IDs. [file gb-2009-10-6-r70-S5.doc]

**Additional data file 5: Detailed legend (including gene ID) of the predicted general metabolism and solute transport in *T. gammatolerans*.**

**(A) Modified Embden-Meyerhof glycolytic pathway**: (ENO, tg0077) enolase; (F1,6BP) fructose 1,6-bisphosphate; (FBA, tg1754) fructose 1,6-biphosphate aldolase; (F6P) fructose 6 phosphate; (G6P) glucose 6 phosphate; (GAP) glyceraldehyde 3 phosphate; (GAPDH, tg1667) glyceraldehyde 3 phosphate deshydrogenase; (GAPOR, tg0122) glyceraldehyde 3 phosphate ferredoxin oxidoreductase; (GLK, tg0818) glucokinase; (2PG) 2 phosphoglycerate; (3PG) 3 phosphoglycerate; (PC, tg1753) pyruvate carboxylase, (PCK, tg1771) phosphoenolpyruvate carboxykinase; (PEP) phosphoenolpyruvate; (PFK, tg1465) 6-phosphofructokinase; (PGI, tg1078) glucose 6-phosphate isomerase; (PGK, tg1318) phosphoglycerate kinase; (PGM, tg0813) phosphoglucomutase; (PGP) 2,3 bisphosphoglycerate; (PPS, tg1043) phosphoenolpyruvate synthase; (PYK, tg1550) pyruvate kinase.

**(B) Pyruvate degradation**: (ADP-ACS, ACSI: tg1201/tg1288, ACSII: tg0426/tg0742, tg0286, tg0741) ADP-forming acetyl-CoA synthetases; (AMP-ACS, tg0230) AMP-forming acetyl-CoA synthetase; (AT, tg1077, tg1590, tg1331, tg1838, tg1056) aminotransferases; (CoA) coenzyme A; (POR, tg0261, tg0262, tg0263, tg0267) pyruvate:ferredoxin oxidoreductase.

**(C) Pentose phosphate synthesis and carbon dioxide fixation**: (APRT, tg1947) adenine phosphoribosyltransferase; (CooC, tg0822) a CooS accessory protein; (CooF, tg0823) electron transfer protein; (CooS, tg0824) carbon monoxide dehydrogenase; (DeoA, tg1786) AMP phosphorylase; (HPS/PHI, tg1891) bifunctional D-arabino 3-hexulose-6-phosphate formaldehyde lyase/phosphohexuloisomerase; (PRPP) 5-phosphoribosyl 1-pyrophosphate; (PRPPS, tg2049) PRPP synthase; (RBPI, tg1633) ribose 1,5 bisphosphate isomerase; (RPI, tg1781) ribose 5-phosphate isomerase, (Rubisco, tg1751) ribulose 1,5-biphosphate carboxylase/oxygenase.

**(D) Pseudo TCA cycle**: (FH, tg0318-tg0319) fumarate hydratase; (FRD, tg0620) fumarate reductase; (MDH, tg1608) malate deshydrogenase; (SCS, tg0218, tg0742) succinyl-CoA synthetase.

**(E) Amino acid degradation**: (GDH, tg1822/tg1823) glutamate deshydrogenase; (KGOR, tg1375, tg1376, tg1377, tg1379, tg1380, tg1381, tg1382) ketoglutarate:ferredoxin oxidoreductase; (IOR, tg0427, tg0428, tg1129, tg2042) indolepyruvate ferredoxin oxidoreductase; (VOR, tg0264, tg0265 and tg0266) 2 oxoisovalenate ferredoxin oxidoreductase.

**(F) Oxygen and reactive oxygen species detoxication**: (SOR, tg1216) superoxide reductase.

**Miscellaneous**: (Cyt bd, tg1232/tg1233) cytochrome bd; (E-4-P) erythrose 4 phosphate; (tg0066, tg0067, tg0068) F_420_-reducing dehydrogenase subunits; (Fdred) reduced ferredoxin; (Fdox) oxidized ferredoxin; (Mbc1, tg0048-tg0054) membrane-bound complex 1; (Mbc2, tg1241-tg1249) membrane-bound complex 2; (Mbh, tg0034-tg0047) membrane-bound hydrogenase complex; (Mbx, tg0703-tg0715) membrane-bound oxydoreductase complex; (Mhy1, tg0241-tg0247) formate hydrogene lyase 1; (Mhy2, tg0056-tg0065) formate hydrogene lyase 2; (NSR, tg1050) NAD(P)H elemental sulfur oxidoreductase; (S^o^) elemental sulfur; (TK, tg1599/tg1600) transketolase; (Xu-5-P) xylulose 5 phosphate.

The transporters are grouped by substrate specificity:

**(i)** **anions** : (eriC, tg0433) Cl^-^ channel protein; (PitA, tg0119) Na^+^/phosphate symporter; (pstABCS, tg0204, tg0206, tg0207, tg0208) ABC-type phosphate transporter.

**(ii)** **amino acids/dipeptides** : (AGCS, tg0308) probable Na^+^/alanine transport protein belonging to the AGCS family; (AAT, tg0091-95) additional amino acid ABC transporter; (App/Opp, tg0152, tg0331-335, tg0383-385, tg0872-876) ABC-type dipeptide/oligopeptide transporter; (Aro, tg1321) aromatic amino acid transporter; (Cat-1, tg1855) cationic amino acid transporter; (GltT, tg1756) H^+^/sodium-glutamate symporter; (PutP, tg0963) sodium/proline symporter, (Neu, tg1060) small neutral amino acid transporter.

**(iii)** **cations** : (cbiOQ, tg1802/tg1801) subunits of cobalt transport system; (Fe^3+^, tg1014, tg1015, tg1016) additional ABC-type Fe^3+^ transport system; (FeoAB, tg1104, tg1105) Fe^2+^ transporter; (FbpABC, tg1704, tg1705, tg1706) ABC-type Fe^3+^ transporter; (FepBCD, tg0819, tg0820, tg0821) ATP-type Fe^3+^ transporter; (FocA, tg0055) formate/nitrate transporter; (Kch, tg0129) K^+^ channel; (MgtE, tg0739, tg0738) divalent cation transporter; (NatAB, tg1121, tg1122) ABC-type Na^+^ efflux pump; (NhaC, tg1715) Na^+^/H^+^ antiporter; (SNF, tg1707) sodium-dependent transporter; (TrkAH, tg0857, tg0285) Trk-type K^+^ transporter; (ZnuABC; tg1644, tg1090, tg1089) ABC-type Mn^2+^/Zn^2+^efflux pump.

**(iv)** **heavy metal or drug**: (ABC-2 multidrug, tg1451, tg1487-88, 1699) putative ABC-type multidrug transporter; (ArsAB, tg2109, tg0945) arsenical pump membrane protein; (DMT, tg0337, tg0342, tg0351, tg0216, tg0415) drug/metabolite transporter; (MATE, tg1034) multi antimicrobial extrusion protein; (RND, tg0080) RND superfamily putative drug exporter; (ZupT, tg0867) heavy-metal transporter from the ZIP family.

**(v)** **carbohydrates** : (MalEFGK, tg0600, tg0601, tg0602, tg0604) ABC-type maltodextrin transporter.

**(vi)** **unknown** : (MscS, tg1007, tg1259) mechanosensitive ion channel; (MFS, tg2011, tg1443, tg1358) transporters related to major facilitator superfamily; (Trap-type transporter, tg1892, tg1894) tripartite ATP-independent periplasmic transporter.
