# Supplementary material for: Genome analysis and genome-wide proteomics of Thermococcus gammatolerans, the most radioresistant organism known amongst the Archaea
Source: Genome Biol. 2009 Jun 26;10(6):R70. doi: 10.1186/gb-2009-10-6-r70 (PMC2718504; doi:10.1186/gb-2009-10-6-r70)
Supplement: Additional data file 7 — Protein sequence alignments of the putative additional MhyI subunits. [file gb-2009-10-6-r70-S7.ppt]

## Slide 1
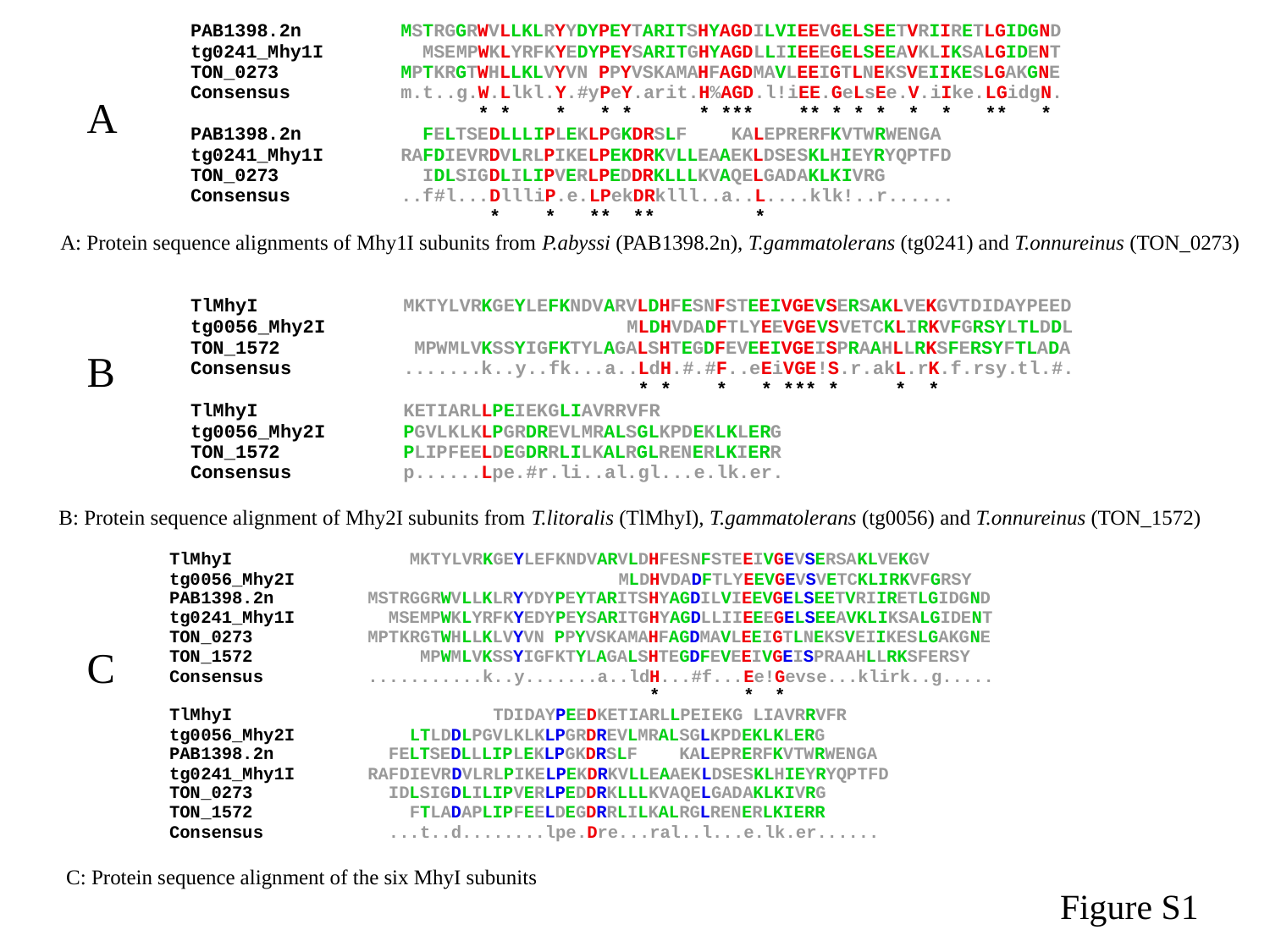

A
A: Protein sequence alignments of Mhy1I subunits from P.abyssi (PAB1398.2n), T.gammatolerans (tg0241) and T.onnureinus (TON_0273)
B
B: Protein sequence alignment of Mhy2I subunits from T.litoralis (TlMhyI), T.gammatolerans (tg0056) and T.onnureinus (TON_1572)
C
C: Protein sequence alignment of the six MhyI subunits
Figure S1
